# Ultra-compact MXene fibers by continuous and controllable synergy of interfacial interactions and thermal drawing-induced stresses

**DOI:** 10.1038/s41467-022-32361-6

**Published:** 2022-08-05

**Authors:** Tianzhu Zhou, Yangzhe Yu, Bing He, Zhe Wang, Ting Xiong, Zhixun Wang, Yanting Liu, Jiwu Xin, Miao Qi, Haozhe Zhang, Xuhui Zhou, Liheng Gao, Qunfeng Cheng, Lei Wei

**Affiliations:** 1 School of Electrical and Electronic Engineering, Nanyang Technological University, Singapore, 639798 Singapore; 2 School of Chemistry, Key Laboratory of Bio-inspired Smart Interfacial Science and Technology of Ministry of Education, Beijing Advanced Innovation Center for Biomedical Engineering, Beihang University, Beijing, 100191 China; 3 School of Transportation Science and Engineering, Beihang University, Beijing, 100191 China; 4 School of Materials Science and Engineering, Zhengzhou University, Zhengzhou, 450001 China

**Keywords:** Mechanical properties, Electronic devices, Mechanical engineering, Two-dimensional materials, Design, synthesis and processing

## Abstract

Recent advances in MXene (Ti_3_C_2_T_*x*_) fibers, prepared from electrically conductive and mechanically strong MXene nanosheets, address the increasing demand of emerging yet promising electrode materials for the development of textile-based devices and beyond. However, to reveal the full potential of MXene fibers, reaching a balance between electrical conductivity and mechanical property is still the fundamental challenge, mainly due to the difficulties to further compact the loose MXene nanosheets. In this work, we demonstrate a continuous and controllable route to fabricate ultra-compact MXene fibers with an in-situ generated protective layer via the synergy of interfacial interactions and thermal drawing-induced stresses. The resulting ultra-compact MXene fibers with high orientation and low porosity exhibit not only excellent tensile strength and ultra-high toughness, but also high electrical conductivity. Then, we construct meter-scale MXene textiles using these ultra-compact fibers to achieve high-performance electromagnetic interference shielding and personal thermal management, accompanied by the high mechanical durability and stability even after multiple washing cycles. The demonstrated generic strategy can be applied to a broad range of nanostructured materials to construct functional fibers for large-scale applications in both space and daily lives.

## Abstract (web summary)

Forming compact layered nanostructures is key to achieving continuous MXene fibers with electrical and mechanical properties. Here, authors demonstrate ultra-compact high-performance MXene fibers via a controllable synergy of interfacial interactions and thermal drawing-induced stresses.

## Introduction

Fibers, as the basic building blocks of textiles, actively engage in a wide range of our daily activities, including health management^1^, human-computer interaction^2^, movement monitoring^3^, soft robotics^4^, disease prevention^5^, and many more. Targeting on achieving fibers with high mechanical and electrical performance, various families of conductive materials have been the main research focus, ranging from carbon-based materials, metal-based materials, to conductive polymers-based materials^6–10^. Among all the accessible materials, MXenes, as an emerging class of 2D inorganic compounds, are emerging yet promising materials for developing fiber-based devices, owing to their combined superior mechanical^11^, electrical^12^, and electromagnetic properties^13^. MXene (Ti_3_C_2_T_*x*_) nanosheets can be prepared into high-performance nanocomposites due to their surface terminated moieties (T_*x*_), such as -OH, -O, and -F^14^. Many studies have been carried out to fabricate MXene fibers based on MXene nanosheets by various processes, including wet spinning^15^, coating^16^, electrospinning^17^, and biscrolling method^18^. MXene fibers have therefore been successfully fabricated with desired electrical conductivity and mechanical properties (such as MXene/rGO^19^, MXene/cellulose nanofibrils^20^, Kevlar/MXene^21^, and nylon/MXene^22^). However, both the electrical conductivity and mechanical properties of MXene nanosheets are not fully utilized in the form factor of fiber, mainly due to the inherently loose layers caused by the structure defects (voids and wrinkles of MXene nanosheets) and poor interlayer interactions between MXene nanosheets^23,24^. Continuous efforts have been proposed to form compact layered structures between MXene nanosheets, mainly using wet spinning^25,26^. Despite the enhanced mechanical and electrical performance, the major challenge remains to reach a balance between electrical conductivity, strength, and toughness, mainly due to the difficulties to further compact the loose MXene layers. Thus, to fundamentally address this challenge, a process that can achieve ultra-compact, continuous, and long MXene fibers in a controllable manner is highly needed, aiming to reveal the full potential of MXene.

After fibers with high mechanical and electrical performance are achieved, they can be used to construct functional textiles for large-scale coverage such as on the human body^27,28^. However, performance degradation of these fibers is the common reason to limit their long-term usage, due to the fact that they are fully exposed to the environment and skin^29^, suffer from physical impact applied directly on them caused by body movements^30,31^, as well as being sensitive and fragile to against routine maintenance such as washing and drying. To solve these issues, an effective method is to form a protective layer on the outer surfaces of these fibers. Achieving this not only requires additional process steps, but also brings up uncertainties on controlling the interaction between the resulting fibers and coatings^32^. Furthermore, it is necessary to conduct an accurate assessment on the properties of flexible fibers with protective layers for the consideration of practical scenarios.

Herein, we demonstrate a continuous and controllable route to fabricate ultra-compact MXene fibers with high electrical conductivity, strength, and toughness. To start, interfacial interactions in the wet spinning enable the transfer from MXene nanosheets to compact MXene fibers. Then, these fibers are continuously fed into a polymer tube in thermal drawing, resulting in (1) ultra-compact MXene fibers enabled by the drawing-induced controllable stresses and (2) in-situ generated protective layer. Thanks to such a synergy of interfacial interactions and thermal drawing-induced stresses, the resulting ultra-compact MXene fibers with protective layers not only offer remarkable tensile strength of 585.5 ± 2.1 MPa and ultra-high toughness of 66.7 ± 5.0 MJ m^−3^, but also exhibit high electrical conductivity of 8802.4 ± 30.8 S cm^−1^ and excellent long-term mechanical durability and stability. After constructing large-scale MXene textiles using these fibers, two representative applications are achieved. One is for electromagnetic interference shielding with a high shielding efficiency of ~57 dB and ~87.8% performance retention after 5 × 10^4^ bending cycles. The other one is for electrothermal effect with the demonstration of a textile generating the heat with the remarkable deformation stability. The demonstrated synergy of interfacial interactions and thermal drawing-induced stresses provides broad interests for achieving high-performance fibers over a wealth of nanostructured functional materials.

## Results

**Fig. 1:**
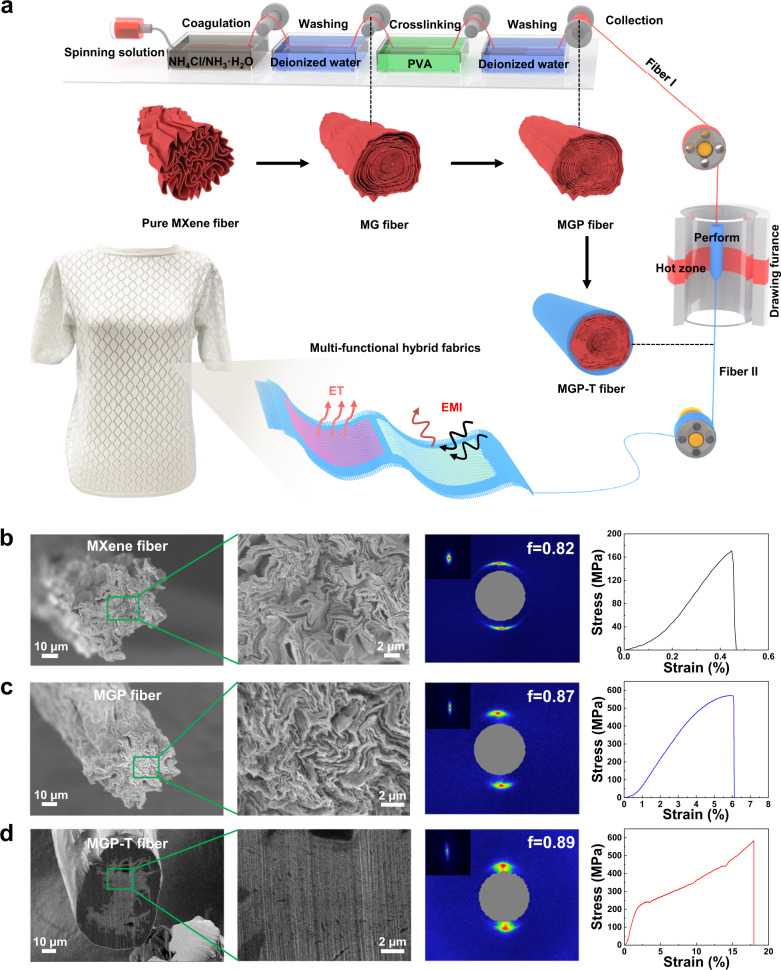
Fabrication flow to achieve ultra-compact MXene fibers. **a** Fabrication of MGP-T fiber via continuous wet spinning and thermal drawing, and the formation of MXene-based textiles. SEM cross-sections of MXene fibers with the WAXS/SAXS patterns and stress-strain curves: **b** Pure MXene fibers; **c** MGP fibers after interfacial interactions; **d** MGP-T fibers after thermal drawing. The results show that the MGP-T fibers offer excellent mechanical properties with high alignment and low porosity, compared to pure MXene and MGP fibers.

The flexible MXene fibers with ultrahigh compactness are fabricated by a continuous and controllable synergy of wet spinning with interfacial interactions and thermal drawing-induced stresses, as schematically illustrated in Fig. 1a. Then, large-area multi-functional fabrics can be constructed by the resulting fibers to engage applications, including electromagnetic interference (EMI) shielding and thermal management based on the electrothermal (ET) effect. Compared to the pure MXene fibers with the poor interfacial interaction, the resulting MXene fibers (MGP) from the first step of wet spinning achieve the increased orientation orders (*f*) from ~0.82 to ~0.87, and low porosity due to the synergistic interfacial interactions, resulting in the enhancement of the tensile strength from 167.1 ± 7.2 (mean ± standard deviation) (coefficient variation, CV, 0.04) MPa to 565.2 ± 5.2 (0.01) MPa with the toughness from 0.4 ± 0.1 (0.25) MJ m^−3^ to 19.2 ± 0.7 (0.04) MJ m^−3^ (Fig. 1b, c). After experiencing the stresses induced by the second step of thermal drawing, the ultra-compact MXene fibers (MGP-T) are achieved with the significantly promoted *f* of ~0.89, accompanied by the further enhancement of alignment and reduction of voids. As a result, the obtained MGP-T fibers with an in-situ generated protective layer offer the highest strength of 585.5 ± 2.1 (0.01) MPa and toughness of 66.7 ± 5.0 (0.07) MJ m^−3^ (Fig. 1d).

### Synthesis and characterization of MXene fibers via wet spinning

To start, MXene (Ti_3_C_2_T_*x*_) nanosheets were prepared by etching and shaking from preliminary Ti_3_AlC_2_ and accordion-like MXene (Supplementary Fig. 1). The obtained MXene nanosheets have a lateral size of ~10 μm (Supplementary Fig. 2a, b) with a thickness of ~1.5 nm according to the scanning electron microscope (SEM) and atomic force microscopy (AFM) images (Supplementary Fig. 2c, d). Moreover, the exfoliated MXene nanosheets have the expected high crystallinity hexagonal structures without defects, confirmed by the high-resolution transmission electron microscopy (HR-TEM) images and the selected area electron diffraction patterns (Supplementary Fig. 3)^33^. In addition, X-ray diffraction (XRD) patterns demonstrate the complete etching of the Al layer without 104 and 105 peaks for Ti_3_AlC_2_ pattern (Supplementary Fig. 4)^34^, indicating that the MXene nanosheets are successfully prepared from Ti_3_AlC_2_.

**Fig. 2:**
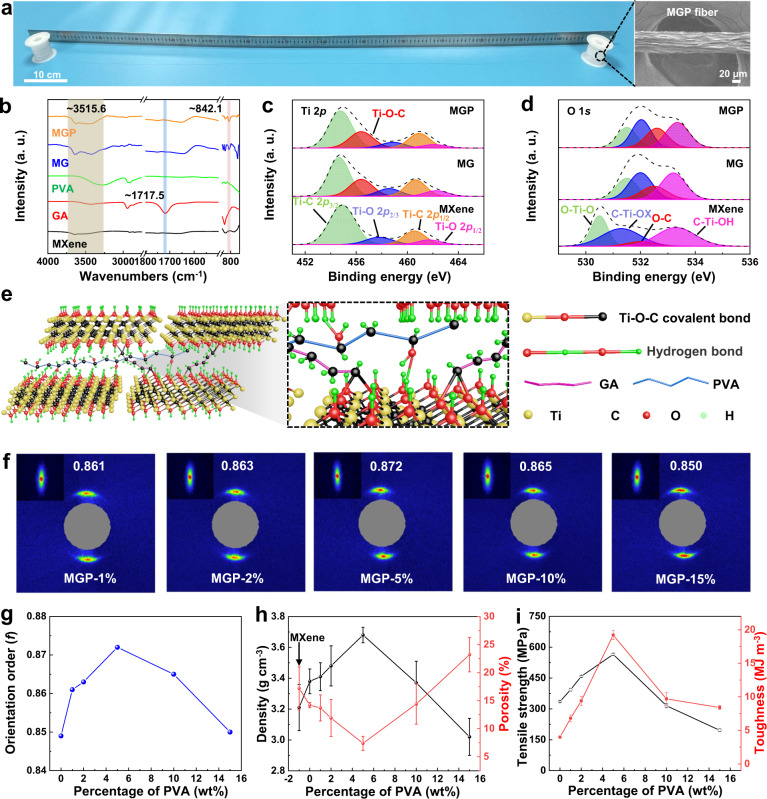
Interfacial interactions and mechanical characterization of MXene fibers via wet-spinning. **a** Photograph of several meter-long MGP fibers with the axial SEM morphology. **b** FTIR spectra of the obtained fibers. **c** Ti 2*p* spectra and **d** O 1*s* spectra of pure MXene, MG, and MGP fibers. **e** Structure graph of the covalent bond between MXene nanosheets and GA, and hydrogen bond between MXene nanosheets and PVA. **f** WAXS and SAXS (inset) patterns graphs of MGP fibers with different weight percentages of PVA. **g** Corresponding orientation order of MGP fibers with different weight percentages of PVA according to the WAXS patterns. **h** Curves of the density and porosity vs. weight percentage of PVA of MGP fibers. **i** Tensile strength and toughness of MGP fibers with different weight percentages of PVA. All error bars show mean ± standard deviation (SD).

According to the Onsager’s theoretical prediction model^7^, MXene nanosheets in the MXene-glutaraldehyde (GA) spinning solution can present lyotropic liquid-crystalline properties in the range of ~15 mg mL^−1^ to ~30 mg mL^−1^ (Supplementary Fig. 5). The mixing spinning solution also shows appearing birefringence, performing the formation of the liquid-crystalline phase as a result of the local orientation without aggregation. Meanwhile, the spinning solution with a high concentration of 30 mg mL^−1^ forms a viscous ink with a viscosity of ~4.1 kPa s without aggregates (Supplementary Fig. 6a). Similar to most complex fluid systems with rigid polymer chains, the viscosity of MXene nanosheets in spinning solutions decreases with an increasing shear rate, and increases with an increasing concentration^35^. Moreover, the shear stress of the spinning solution decreases sharply at the initial stage and then gradually increases with the shear rate (Supplementary Fig. 6b). It suggests that the randomly oriented MXene nanosheets turn into an ordered state, because of the shear-induced deformation^36^. Furthermore, as shown in Supplementary Fig. 6c, the ratio of the storage modulus to the loss modulus (*G*’/*G*”) of a spinning solution is in the range from 1.86 to 6.01 with the concentration of 30 mg mL^−1^ to 15 mg mL^−1^, acting as an indicator for the spinnability of liquid-crystalline MXene nanosheets colloidal dispersions^32^. Moreover, with the systematic optimization of the concentration of 15 to 50 mg mL^−1^ (Supplementary Figs. 7a, b, 8 and Supplementary Tables 1, 2) and draw ratio of 0.5 to 2.8 (Supplementary Figs. 7c, d, 11, 12 and Supplementary Tables 4, 5), MXene (Ti_3_C_2_T_*x*_) nanosheets with glutaraldehyde solution with the concentration of 30 mg mL^−1^ can be extruded to form the fibers with high mechanical and conductive properties at the draw ratio of 2.8 due to the high orientation and low porosity (Supplementary Figs. 9, 10, 13, 14 and Supplementary Tables 3, 6). In this process, MXene nanosheets are initially crosslinked with GA at a concentration of 30 mg mL^−1^ to form MXene-GA (MG) fibers at the draw ratio of 2.8, and sequentially crosslinked with polyvinyl alcohol (PVA) to fabricate several meters long MXene-GA-PVA (MGP) fibers with a diameter of ~60 μm by wet spinning (Fig. 2a and Supplementary Movie 1).

Fourier transform infrared spectroscopy (FTIR) spectra show a new peak at ~842.1 cm^−1^ for the MG and MGP fibers, indicating the formation of Ti-O-C bond between MXene nanosheets and GA molecules^37,38^ (Fig. 2b and Supplementary Fig. 15). Moreover, the disappearance of peak of ~1717.5 cm^−1^ for the aldehyde group (-CHO) of GA molecules also confirms the formation of the Ti-O-C bond. Obviously, the peak of -OH of MXene nanosheets shifts from ~3651.2 cm^−1^ to the low wavenumber of ~3515.6 cm^−1^ and becomes wider, due to the hydrogen bond between -OH of MXene nanosheets and -OH of PVA molecules. Furthermore, X-ray photoelectron spectroscopy (XPS) spectra exhibit no existence of Al element for MXene nanosheets in pure MXene, MG, and MGP fibers compared to that of Ti_3_AlC_2_, suggesting the successful preparation of MXene nanosheets (Supplementary Fig. 16). In addition, as shown in Fig. 2c, a new peak at ~456.4 eV for MGP and MG fibers is testified the formation of Ti-O-C between MXene nanosheets and GA, compared with pure MXene fibers according to Ti 2*p* spectrum^38^. The atomic percentage for O-C for MG and MGP fibers is increased to 16.4% and 22.0%, compared to pure MXene fibers of 6.2% in O 1*s* spectra (Fig. 2d and Supplementary Table 7), indicating the formation of Ti-O-C bond between MXene nanosheets and GA, and hydrogen bond between MXene nanosheets and PVA. Hence, compact MXene fibers are fabricated via interfacial interactions, which are firstly crosslinked with GA via Ti-O-C bond of nucleophilic substitution (Supplementary Fig. 17), and then crosslinked with PVA via hydrogen bond between MXene nanosheets and PVA, as explained in Fig. 2e.

### Mechanical properties of MXene fibers via wet spinning

As a result of the interfacial interactions, the alignment of MXene fibers is significantly enhanced, and the porosity is largely reduced, according to the wide-angle X-ray scattering/small-angle X-ray scattering (WAXS/SAXS) patterns. As shown in Supplementary Figs. 18, 19, when MXene nanosheets are crosslinked with GA via Ti-O-C covalent bond, the orientation orders (*f*) of the obtained MG fibers increase to ~0.85 with ~5 wt% GA (TGA results, Supplementary Fig. 21), and decrease to ~0.72 with 20 wt% GA compared with the orientation order of ~0.82 for pure MXene fibers (Supplementary Table 8). Moreover, the porosity of fibers reduces to 14.2 ± 0.45%, with ~5 wt% GA lower than that of 17.2 ± 3.84% for pure MXene fibers. This is because that more GA molecules are hindered in the inter-layer, resulting in the appearing wrinkles and voids according to the increasing intensity of SAXS patterns (Supplementary Fig. 20a). After PVA is cross-linked with MXene nanosheets into MGP fibers, the *f* is enhanced to ~0.87, higher than that of pure MXene fibers and MG fibers (Fig. 2f, g and Supplementary Table 9). The orientation orders of MGP fibers are increased with the increment of PVA up to 5%, while decreased to ~0.85 with the further increment of PVA. Moreover, the porosity is significantly reduced to 7.4 ± 1.27% to achieve a high density of ~3.68 g cm^−3^ via the hydrogen bond of PVA (Fig. 2h). PVA with more than 5 wt% may lead to the increase of wrinkles of MXene nanosheets and voids between MXene nanosheets, which is testified by the increasing intensity of SAXS patterns for MGP fibers (Supplementary Fig. 20b). Therefore, the compact MGP fibers are achieved with low porosity than MG and MXene fibers, according to the decreased intensity of SAXS patterns (Supplementary Fig. 20c).

Due to the high alignment and low porosity achieved by the interfacial interactions, the prepared MG fibers exhibit a higher tensile strength of 335.6 ± 3.8 (0.01) MPa with the toughness of 4.0 ± 0.12 (0.03) MJ m^−3^, compared to that of 167.1 ± 7.2 (0.04) MPa and 0.4 ± 0.1 (0.25) MJ m^−3^ for pure MXene fiber via Ti-O-C covalent bond between MXene nanosheets and GA molecules (Supplementary Figs. 22a, 23 and Table 10). When the GA weight percentage is more than 5 wt%, both tensile strength and toughness decrease for MG fibers, which is attributed to hindering stress transfer with a higher weight percentage of GA between MXene nanosheets. Supplementary Fig. 22b and Table 11 show that the conductivity of MG fibers decreases from 11,360.4 ± 227.2 S cm^−1^ to 4536.4 ± 30.8 S cm^−1^, due to the introduction of non-conductive GA molecules into the MXene nanosheets. However, MG fibers with 5 wt% GA can still keep a high conductivity of 9860.6 ± 117.5 S cm^−1^, because of the high alignment and low porosity of MG fibers. After being subsequently crosslinked with PVA molecules, the tensile strength and toughness are further enhanced to 565.2 ± 5.2 (0.01) MPa and 19.2 ± 0.7 (0.04) MJ m^−3^, as a result of the significant improvement of alignment and decrease of porosity via the synergistic interfacial interactions of the covalent bond and hydrogen bond, shown in Fig. 2i and Supplementary Fig. 24 and Table 12. In addition, the mechanical properties of MGP fibers are weakened by increasing the weight percentage more than ~5 wt% PVA due to the hindering effect. Although the conductivity of MGP fibers slightly decreases with the increment of non-conductive PVA, MGP fiber with ~5 wt% PVA still offers a high conductivity of 8110.4 ± 115.6 S cm^−1^, thanks to the compactness via synergistically interfacial interactions (see Supplementary Fig. 25 and Table 13)^6,39^. The achieved MGP fibers with the compact morphology (Supplementary Fig. 27) exhibit higher performance than pure MXene and MG fibers (Supplementary Fig. 26).

### In-situ characterization of MXene fibers being heated

**Fig. 3:**
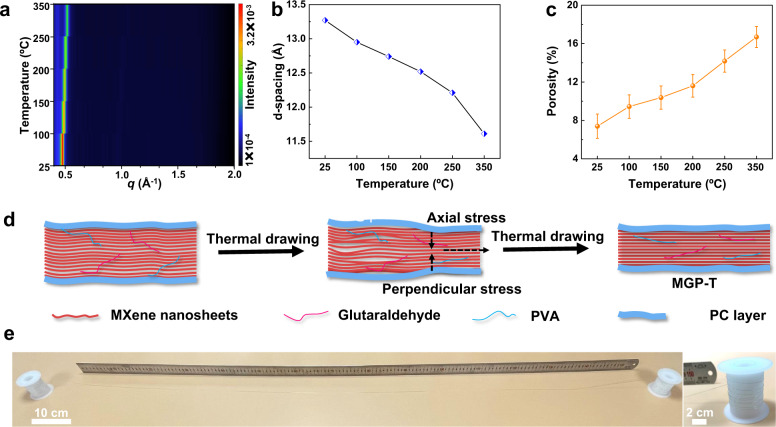
In-situ XRD patterns and fabrication of MGP-T fibers by thermal drawing. **a** In-situ XRD patterns graph of MGP fiber. **b** Curve of d-spacing vs. temperature according to in-situ XRD patterns. **c** Porosity of MGP fibers when being heated. **d** Mechanism schematic diagram to fabricate MGP-T fibers from MGP fibers with axial stress and perpendicular stress during thermal drawing. **e** Photograph of several meters long MGP-T fibers. All error bars show mean ± SD.

Then, the heating process induced property change of MGP fibers was studied at various heating temperatures by in-situ XRD patterns. When an MGP fiber is heated from 25 ºC to 350 ºC, the 002 pattern is shifted to high *q* with the d-spacing of MXene nanosheets down to 11.61 Å, according to the in-situ XRD patterns (Fig. 3a, b and Supplementary Fig. 28). The results suggest that the d-spacing of MXene nanosheets is decreased with the increase of temperature^37^. Moreover, as shown in Fig. 3c, the porosity increases sharply from 7.4 ± 1.27% to 16.7 ± 1.10% when increasing the heating temperature, indicating that the voids and wrinkles between MXene nanosheets are generated when heating (Supplementary Fig. 29a). In addition, the in-situ SAXS patterns were also conducted to study the heating process of fibers, as presented in Supplementary Fig. 29b, c. The increasing intensity of SAXS patterns for MGP fibers confirms the generation of voids when increasing annealing temperature. The voids are mainly generated by removing intercalated water and partial hydroxyl surface terminations at elevated temperatures^40^.

### Fabrication of ultra-compact MXene fibers via thermal drawing

To further enhance the compactness of MXene fibers, thermal-drawing induced physical compression and stretch were developed. More importantly, such a continuous drawing process can protect the resulting ultra-compact MXene fibers with an in-situ formed polymer layer, resulting in a packaged fiber device to directly engage various applications. In the process of thermal drawing, a hollow polycarbonate (PC) tube passed through the hot zone of the drawing furnace, while an MGP fiber was continuously fed into the PC tube from the top of it (Fig. 1a). As shown in Fig. 3d, the MGP fibers in the PC tube generated voids and wrinkles when the whole tube entered the hot zone at the central temperature of ~200 ºC, which was proved by the in-situ XRD and SAXS patterns. However, when thermal drawing started, the generated voids and wrinkles were significantly reduced by the drawing-induced stresses, including both axial stress and perpendicular stress. Meanwhile, the MGP fibers with an outer protective layer (MGP-T) were formed in-situ to protect the resulting ultra-compact MXene fibers. As a result, several meters long MGP-T fibers were successfully fabricated by this strategy with a diameter of ~75 μm (Fig. 3e, Supplementary Fig. 30 and Movie 2).

**Fig. 4:**
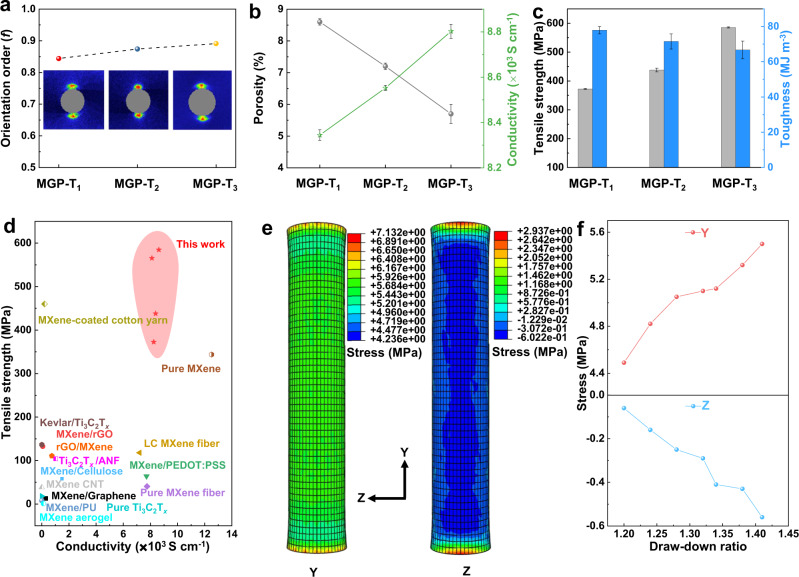
Mechanical properties and mechanism of MGP-T fibers by thermal drawing. **a** Orientation order of MGP-T fibers fabricated by various draw-down ratios according to the WAXS patterns. Curves of the porosity and conductivity (**b**) and tensile strength and toughness (**c**) of MGP-T fibers. **d** Comparison of tensile strength and conductivity of the obtained ultra-compact MXene-based fibers with the reported MXene-based fibers. Stress distribution of the simulation (**e**) and the Y/Z stress curves at the increment of draw-down ratio (**f**) according to finite element analysis. All error bars show mean ± SD.

Using this controllable and continuous process, various MGP-T fibers were fabricated by increasing the draw-down ratio of pull to feed speed of 1.26, 1.34, and 1.41, forming MGP-T_1_, MGP-T_2_, and MGP-T_3_, respectively. As a result of the induced stress parallel to the axial direction in the thermal drawing process, the alignment of MGP fibers is enhanced from ~0.84 to ~0.89 when increasing the draw-down ratio, according to the WAXS patterns presented in Fig. 4a, Supplementary Fig. 31, and Table 14. Simultaneously, the stress perpendicular to the axis effectively compresses the MGP fibers and reduces the porosity from 8.6 ± 0.1% to 5.7 ± 0.3% during the thermal drawing (Fig. 4b). Furthermore, as shown in Supplementary Fig. 32, the SEM images with the EDS mapping of the cross-section for MGP-T fibers clearly show that the fibers become more compact with low porosity when increasing the draw-down ratio. In addition, the HR-TEM images of MGP-T fibers confirm the high alignment of alternative MXene layers and PVA/GA polymer layers (Supplementary Fig. 33). Therefore, the ultra-compact MGP-T fibers with in-situ generated protective layer are achieved with a significant enhancement of the alignment and reduction of the porosity, induced by the controllable thermal drawing stresses^41^. As a result, the conductivity of the inner ultra-compact MXene fibers is promoted from 8344.5 ± 23.4 S cm^−1^ to 8802.4 ± 30.8 S cm^−1^ in Fig. 4b and Supplementary Table 15. Meanwhile, MGP-T fibers exhibit an ultra-high tensile strength of 585.5 ± 2.1 (0.01) MPa and toughness of 66.7 ± 5.0 (0.07) MJ m^−3^ due to the promoted alignment and reduction of porosity during the thermal drawing process with increasing stresses (Fig. 4c and Supplementary Fig. 34, Table 16).

Moreover, the cyclic loading behavior of MXene-based fibers was also conducted with various loading stress (Supplementary Fig. 35). When applied the MXene, MG, MGP, and MGP-T fiber with the loading stress of 150 MPa after 5000 cycles of loading, the MGP-T fiber showed a high electrical conductivity retention rate of 98.9%, and even 93.5% at the loading stress of 300 MPa after 5000 cycles (Supplementary Table 17). In addition, the MGP-T fiber performed an electrical conductivity retention rate of 85.4% when applied loading of 350 MPa after 5000 cycles. These high-performance results are due to the ultra-compact structure of MGP-T fiber via the synergy of interfacial interactions and thermal drawing-induced stresses. As summarized in Fig. 4d and Supplementary Table 18, the obtained ultra-compact MGP-T fibers offer the highest performance on both tensile strength and conductivity, compared with the reported MXene fibers without protective layer^7,15,19–21,23,25,26,36,42–46^. Especially, the toughness of the ultra-compact MGP-T fibers reaches ~66.7 MJ m^−3^, higher than that of the reported MXene-based fibers, graphene-based fibers, and CNT fibers via different preparation strategies (Supplementary Fig. 36 and Supplementary Tables 18 and 19).

### Finite element analysis for thermal drawing process

With the combined effects of the drawing stress parallel to the axial and compressing stress perpendicular to the axial of fibers induced by the thermal drawing, MGP-T fibers with high alignment and low porosity have been achieved, which further enhance their mechanical and electrical properties. In order to understand the role of drawing-induced stresses in the formation of ultra-compact MGP-T fibers, finite element analysis was conducted by the Abaqus software. During the entire procedure of thermal drawing, the compressing effect existed when the PC hollow tube just started to contact the MGP fibers with a diameter of ~60 μm. Therefore, the compressed mechanical behavior of the PC hollow tube was studied when it passed through the drawing furnace (Supplementary Fig. 37a). Here, a PC hollow tube model with an inner diameter of 0.06 mm, an outer diameter of 0.08 mm, and 0.50 mm long was constructed to study the mechanical behavior of PC hollow tube (see Supplementary Fig. 37b). When the drawing force was applied on the bottom of the PC hollow tube during the thermal drawing, the PC model was stretched with the reduction of the diameter of the PC hollow tube (Supplementary Movie 3). According to the simulation results as shown in Fig. 4e, the stress parallel to the axial (Y) is generated accompanied with compressing stress perpendicular to the axial (Z) of the PC hollow tube. Therefore, the alignment of inner MGP fibers is promoted due to axial stress, while the porosity is reduced because of the perpendicular stress. When increasing the draw-down ratio in simulation, both Y drawing stress and Z compressing stress are increased, according to the results of finite element analysis (Fig. 4f). The increasing stresses further enhance the alignment and reduce the porosity, resulting in ultra-compact MGP-T fibers with high mechanical and electrical properties. These results agree well with the experimental results.

### Large-scale wearable MXene textiles for electromagnetic interference shielding and electrothermal applications

**Fig. 5:**
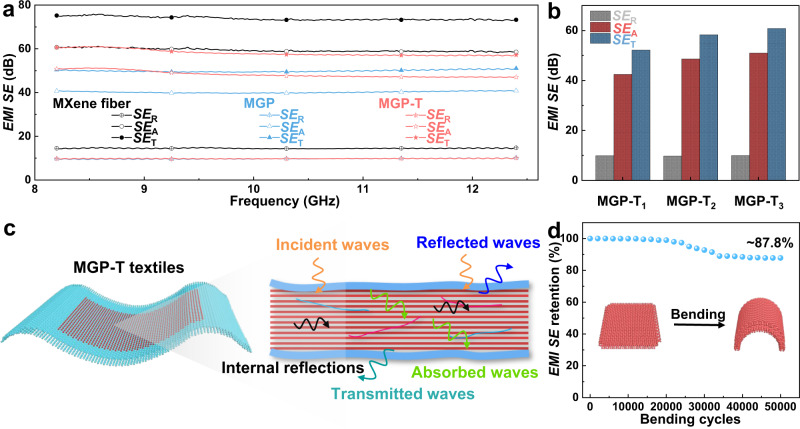
Applications for electromagnetic interference shielding. EMI *SE*_R_, *SE*_A_, and *SE*_T_ of **a** the textiles at the frequency of 8.2 GHz-12.4 GHz and **b** the textiles from MGP-T fibers fabricated via various draw-down ratios at the frequency of 8.2 GHz. **c** The EMI mechanism of the MGP-T textiles. **d** *EMI SE* retention of textile after 5 × 10^4^ bending cycles.

With the rapid development of telecommunication and the ever-increasing usage of portable electronic devices, it would result in serious electromagnetic problems and impact on human beings’ health^40,47^. Attributed to the excellent properties of MXene nanosheets, the obtained ultra-compact MGP-T fibers not only offer high toughness, tensile strength, and electrical conductivity, but also provide potentially helpful EMI shielding performance with high shielding efficiency (*SE*). Figure 5a shows the reflection *EMI SE* (*SE*_R_), absorption (*SE*_A_), and total (*SE*_T_) of textiles based on pure MXene, MGP, and MGP-T fibers in the X-band frequency range of 8.2 GHz–12.4 GHz. The textiles are plain-weaved, as shown in Supplementary Fig. 38. The as-synthesized MGP and MGP-T fiber textiles show the average *SE*_T_ of ~50 dB and ~57 dB, compared to that of pure MXene fiber textile (~74 dB), because the electrical conductivity of MGP and MGP-T fibers is lower than that of pure MXene fibers. However, these results show that the *SE*_A_ (~60 dB, ~47 dB, and ~40 dB) is higher than *SE*_R_ (~15 dB, ~10 dB, and ~10 dB) for pure MXene, MGP fiber, and MGP-T fiber textiles, respectively. Therefore, the EMI shielding is mainly dependent on the absorption mechanism. Furthermore, the *EMI SE* of MGP-T fiber textiles fabricated via increasing draw-down ratios is summarized in Fig. 5b and Supplementary Fig. 39. The MGP-T fiber textiles exhibit a higher *SE*_A_ than *SE*_R_. The *SE*_T_ of MGP-T_1_ changes from ~52 dB to ~61 dB of MGP-T_3_, accompanied by an increment of *SE*_A_ from ~42 dB to ~51 dB at the shielding frequency of 8.2 GHz. It is attributed to the reduction of porosity and enhancement of alignment of MGP-T fibers when applying the high draw-down ratio. However, there is no noticeable increment of *SE*_R_ for MGP-T fiber textiles, as the major EMI mechanism is absorption. Also, the MGP-T fiber textiles show higher *SE*_A_ and *SE*_T_, compared with these of MGP fiber textiles, due to the enhanced compactness of fibers during the thermal drawing procedure. As shown in Fig. 5c, the EMI shielding mechanism of MGP-T fibers can be illuminated as follows^13^: when electromagnetic waves (EMWs) strike the surface of an MGP-T fiber, some EMWs are immediately reflected at the surface because of enormous free electrons of the highly conductive MXene nanosheets. Then, the remaining waves go through the lattice structure of MXene nanosheets, which interact with the high electron density of MXene nanosheets and induce currents to reduce the energy of the EMWs with the ohmic losses. After going through the first layer of MXene, the surviving EMWs could encounter the next MXene barrier layer with the repetition of EMW attenuation. Meanwhile, the second layer as the surface reflects the surviving EMWs and results in multiple internal reflections. Finally, the EMWs are reflected back and forth and then completely absorbed within the compact layers. Therefore, the compact structure provides MGP-T fibers with the superior ability to behave as a multilevel EMI shield. In addition, the textiles based on MGP-T fibers maintain the remarkable bending stability with ~87.8% retention of *EMI SE* performance after 5 × 10^4^ bending cycles (Fig. 5d).

**Fig. 6:**
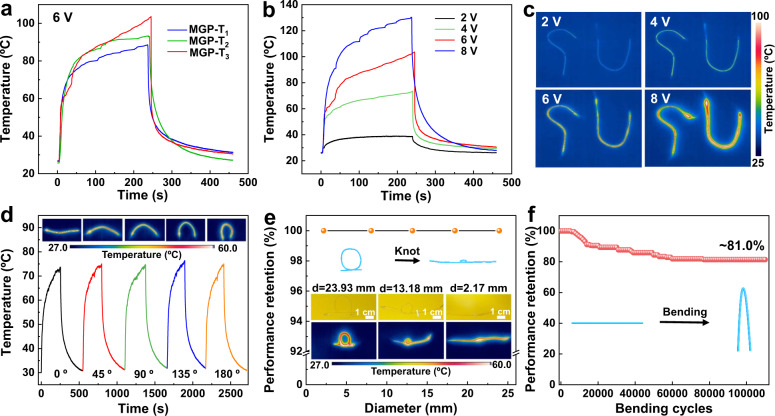
Applications for electrothermal heat management. Temperature-time curves of MGP-T fibers when applied DC voltage of 6 V (**a**) and 2 V-8 V (**b**). **c** Photography of MGP-T fibers with different letter shapes when applied various DC voltage of 2 V-8 V. **d** Temperature-time curves of single MGP-T fibers at the bending angles from 0º to 180º. **e** The temperature performance retention. **f** The temperature performance retention of a single MGP-T fiber after 1.1 × 10^5^ bending cycles from the bending angles of 0º to 180º.

Besides the high EMI shielding performance, the obtained MGP-T fibers also show excellent electrothermal (ET) performance for human thermal management in wearable textiles. Figure 6a shows the temperature-time curves of single MGP-T fibers. When applying the working DC voltage of 6 V, the MGP-T fibers immediately generate thermal energy and reach the equilibrium temperature from ~90 ºC to ~100 ºC (from MGP-T_1_ to MGP-T_3_), owing to the mechanism of the Joule heat^20^. Moreover, after applying different voltages at a broad range of 2 V to 8 V, MGP-T_3_ fibers can generate the heat with the temperature increasing up to ~130 ºC (Fig. 6b), suggesting that the maximum temperature generated by the MGP-T fiber is tunable by applying various DC voltages. These results indicate that MGP-T fibers provide excellent ET behavior for their Joule heating. Furthermore, a single MGP-T fiber performs an excellent cycling life with performance retention of ~99% after 5000 cycles (Supplementary Fig. 40).

In addition, in order to achieve the practical applications of MGP-T fibers for thermal management, fabrics with different shapes of the letters based on MGP-T fibers could effectively generate the heat by varying the applied voltages (Fig. 6c). Moreover, the mechanical durability of the MGP-T fibers needs to be considered for further applications. As shown in Fig. 6d, the mechanical stability of a single MGP-T fiber was conducted when bending at the bending angle of from 0º to 180º. The obtained stable temperature-time curves with the equilibrium temperature are the same as the initial curve, according to the infrared (IR) images. Meanwhile, the MGP-T fibers were availably tied into knots with various diameters from ~23.93 mm to ~2.17 mm, as shown in Fig. 6e. The performance of ET behavior remained stable, evaluated through the IR images. Particularly, the obtained MGP-T fibers could keep the temperature performance retention of ~81% after suffering from ~1.1 × 10^5^ bending cycles (Fig. 6f). Furthermore, compared with other MXene-based materials reported in the literature, our ultra-compact MGP-T fibers performed the highest tensile strength and toughness, and the textile prepared with MGP-T fibers performed the high specific shielding effectiveness per thickness (*SSE*_t_)^13,48,49^ of 4.3 × 10^4^ dB cm^2^g^−1^ and ET performance, as summarized in Supplementary Tables 20, 21.

**Fig. 7:**
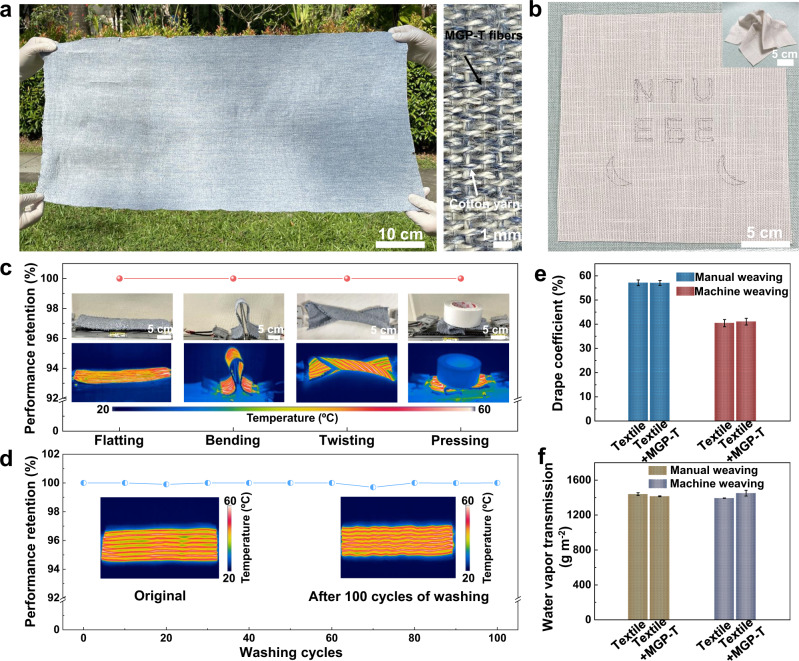
Large-scale wearable MXene textiles. **a** Photograph of a textile (0.8 m by 0.4 m) prepared via machine weaving with MGP-T fibers. **b** Designed patterns woven into the cotton cloth under complex deformations (inset). **c** Deformation stability of the machine weaving textile. **d** Washing durability for a textile after 100 cycles of washing. Drape (**e**) and moisture vapor transmission rate (MVTR) (**f**) performance of manual weaving and machine weaving textiles with the ultra-compact MGP-T fibers. All error bars show mean ± SD.

With the high flexibility of MGP-T fibers (Supplementary Table 22), the textiles with ultra-compact MGP-T fibers were prepared by machine weaving and manual weaving. As a result, the MGP-T fibers were successfully woven into cotton yarn via machine weaving with MGP-T fibers to form a piece of textile (0.8 m by 0.4 m) using one hundred and fifty meters long ultra-compact MGP-T fibers (Fig. 7a and Supplementary Figs. 41, 42). Moreover, 18-meter long ultra-compact MGP-T fibers were fully woven into a piece of cotton cloth with a length of 2.0 meters and width of 0.6 meters by embroidery technique (Supplementary Fig. 43). These results indicate that the ultra-compact MGP-T fibers can enable the large-scale applications, especially acting as wearable functional textiles for thermal management and EMI shielding. Also, some designed patterns were achieved in the cotton cloth using the flexible MGP-T fibers and remained the performance even under complex deformations, indicating their excellent stitchability for various applications (Fig. 7b and inset). Eventually, a sweater woven with several MGP-T fibers could quickly generate the heat with the temperature of ~70 ºC by applying the DC voltages of 4 V (see Supplementary Movie 4) for wearable human thermal management. Moreover, the machine weaving textile by loom performed the remarkable electrothermal performance retention of ~100% at different bending ratios from 1 to 0.1 (Supplementary Fig. 44a, b and Supplementary Movie 5) at the applied DC voltage of 4 V, and even had the retention of ~91.3% after 4 × 10^4^ cycles of bending (Supplementary Fig. 44c). Furthermore, the machine weaving textile showed excellent deformation stability with ~100% electrothermal performance retention under flatting, bending, twisting, and even pressing with a l-kg load (Fig. 7c). These flexibility results of textiles were attributed to the ultra-compact MGP-T fibers with high mechanical properties.

In addition, the washing durability of the machine weaving and manual weaving textiles with ultra-compact MGP-T fibers was evaluated after 100 washing cycles following the ISO 6330 standard, and the obtained ultra-compact MXene fibers were stable and compatible with daily applications (Fig. 7d and Supplementary Fig. 45). Two types of textiles, including manual weaving and machine weaving with MGP-T fibers, were tested for drape behavior, moisture vapor transmission rate (MVTR), and drop absorbency behavior. These textiles showed the drape coefficient of 57.05 ± 1% and 41.1 ± 1.4% compared with the control textiles of 57.12 ± 1.2% and 40.5 ± 1.5% (Fig. 7e), and the water vapor transmission of 1415.41 ± 4.55 g m^−2^ and 1451.63 ± 33.04 g m^−2^ compared with the control textiles of 1439.68 ± 15.49 g m^−2^ and 1393.71 ± 2.41 g m^−2^ (Fig. 7f). In addition, there is no obvious drop absorbency difference between the two types of textiles with and without the ultra-compact MGP-T fibers (Supplementary Table 23). These results indicate that the woven MGP-T fiber in the prepared textiles have negligible effects on the drape, MVTR, and drop absorbency performance. Furthermore, the prepared MXene-based fibers including MGP-T fibers performed no toxicity (Supplementary Fig. 46). The obtained results reveal that the ultra-compact MGP-T fibers are very promising for constructing smart textiles with diverse functionalities and high mechanical durability and stability. As a result, by leveraging on their excellent mechanical and electrical properties, the smart textiles based on the ultra-compact MXene fibers are potentially applied in the personal protection for the electromagnetic interference shielding and electrothermal management in both space and daily lives.

## Discussion

This work demonstrated an effective and continuous strategy to fabricate ultra-compact MXene fibers with an in-situ generated protective layer via the combination of wet spinning and thermal drawing. Due to the interfacial interactions and drawing-induced stresses, the alignment of MGP-T fibers was significantly enhanced together with the effective reduction of porosity of MXene nanosheets. Consequently, the resulting ultra-compact MGP-T fibers showed excellent mechanical and electrical properties. In addition, the MGP-T fibers did not only offer remarkable electromagnetic interference shielding performance, but also performed excellent electrothermal performance with ultra-stable mechanical durability, as well as being washable. Meanwhile, the woven textiles based on MGP-T fibers were constructed to work properly to achieve large-scale applications even under complex deformations. These results demonstrate that the strategy can provide an innovative way to fabricate compact fibers with multifunction, paving the way towards intelligent textiles. More importantly, such a strategy can be generally applied to fabricate high-performance fibers over a wide range of nanostructured functional materials, fundamentally boosting their properties to meet various requirements.

## Methods

### Materials

Ti_*3*_AlC_*2*_ powders (particle size ~ 400 mesh) were obtained from Jilin 11 Technology Co., Ltd. Lithium fluoride, ammonium chloride, ammonium hydroxide solution (28.0–30.0 wt%), and glutaraldehyde solution (25 wt%) were purchased from Sigma-Aldrich Co., Ltd. Polyvinyl alcohol (PVA) with a molecular weight of MW ≈ 130,000 was purchased from Sigma-Aldrich. All materials were used as received. PVA solutions used for wet spinning were prepared by dissolving 15 g of PVA powder in 1 L of 95 °C deionized water under vigorous stirring for 12 hours. Polycarbonate (PC) was purchased from Goodfellow Co., Ltd.

### Characterization

SEM images were collected using a JEOL JSM-7600F Schottky field emission scanning electron microscopes at a voltage of 5 kV. High-resolution transmission electron microscope (HR-TEM) images were obtained using a JEOL 2010HR instrument and an acceleration voltage of 200 kV. AFM images were tested by a Bruker Dimension Icon. TGA results were obtained under nitrogen using a Q500 SDT from Thermal Instruments and a heating rate of 10 K min^−1^ between 35 °C and 800 °C. XRD patterns were conducted using Cu-Kα radiation and an XRD Bruker D8 Advance. The rheological behaviors of the MXene-glutaraldehyde spinning solutions were investigated with a rheometer (Anton Paar MCR 501) under both steady shear and dynamic oscillatory conditions. The viscoelastic properties of the spinning dispersion were measured by measuring the storage and loss modulus as a function of frequency from 0.1 to 100 rad s^−1^. The strain amplitude remained at 0.1% with a gap of 1 mm at 25 °C for the frequency sweep. Polarized optical microscopy (POM) images were recorded to exhibit optical birefringence via Olympus BX51. FTIR spectra were recorded at room temperature with Diamond ATR by an FTIR Frontier from PerkinElmer. XPS spectra were obtained using an XPS Kratos AXIS Supra. Electrical conductivities were tested using a standard two-probe method with a Keithley 2700 source meter. WAXS/SAXS measurements were conducted on a SWAXS Xenocs Nanoinxider, which was equipped with a Cu-Kα source (operated at 30 W) with a beam diameter of 200 to 800 mm on SAXS (200 K) and WAXS (100 K) detectors. In-situ XRD variable temperature tests were also conducted with the Linkam temperature stage by a SWAXS Xenocs Nanoinxider. The cross-section morphology of MGP-T fibers was obtained by Focused Ion Beam (FIB) with the equipment of ZEISS Crossbeam 540 FIB-SEM. Tensile stress-strain curves were measured on 20 mm long, 3 mm wide samples using a SUNS EUT4103X Tester at a loading rate of 0.3 mm min^−1^ with a 10 N sensor at room temperature. The area of fibers was measured by SEM. The results for each fiber were evaluated from the average value of at least three samples.

### Finite element analysis of the thermal drawing

The finite element model was conducted by the commercial software Abaqus 2019 with Abaqus/explicit. During the thermal drawing, the PC tube did not contact the MGP fibers at the hot zone at the central temperature of ~200 ºC until the temperature of ~160 ºC in the drawing furnace, according to the experiments. Here, the compressed mechanical behavior of the PC hollow tube was the focus of research when the PC hollow tube and MGP fiber came into contact until passing through the drawing furnace at the temperature of 160 °C. In the simulation, due to the diameter of ~60 μm for MGP fibers, the PC hollow tube model with an inner diameter of 0.06 mm, an outer diameter of 0.08 mm, and 0.50 mm length was constructed to study the mechanical behavior of PC hollow tube during thermal drawing. The PC hollow tube with the isotropic bulk modulus (*E*) of 0.18 GP, Poison ratio (*υ*) of 0.35. The mechanical property of PC at the temperature of 160 °C was characterized by the DMA instrument (DMA Q800). The fixed boundary conditions were set at the top of the PC hollow tube, while the pulling force was applied at the bottom of the hollow tube. In order to study the mechanical behavior of various draw-down ratios for thermal drawing, increasing the pulling force was used as same as the conditions of the experiments. The material model was defined with isotropic elasticity-plasticity of Eqs. (1) and (2) as follows:

1 $$ \left\{\begin{array}{c}\varepsilon_{11}\\ \varepsilon_{22}\\ \varepsilon_{33}\\ \gamma_{12}\\ \gamma _{13}\\ \gamma_{23}\end{array}\right\}=\left[\begin{array}{cccccc}1/{E} & -{\upsilon }/{E} & -\upsilon /{E} & 0 & 0 & 0\\ -{\upsilon }/{E} & 1/{E} & -{\upsilon}/{E} & 0 & 0 & 0\\ -{\upsilon }/{E} & -{\upsilon }/{E} & 1/{E} & 0 & 0 & 0\\ 0 & 0 & 0 & 1/{G} & 0 & 0\\ 0 & 0 & 0 & 0 & 1/{G} & 0\\ 0 & 0 & 0 & 0 & 0 & 1/{G}\end{array}\right]\left\{\begin{array}{c}\sigma_{11}\\ \sigma_{22}\\ \sigma_{33}\\ \sigma_{12}\\ \sigma_{13}\\ \sigma_{23}\end{array}\right\}$$

2 $$G{{\mbox{=}}}\frac{E}{2\times \left(1{{\mbox{+}}}\upsilon \right)}$$

where *G*, *E*, and *υ* are the shear modulus, Young’s modulus, and Poisson’s ratio, respectively.

### Measurement for electromagnetic interference (EMI) shielding performance

The woven textiles, prepared by three layers of MXene-based fibers, were tested for EMI shielding in a DR-WX rectangular waveguide using the N9917A network analyzer (Agilent Technologies, USA) at the X-band frequency range of 8.2 GHz to 12.4 GHz. The MXene-based fibers were weaved into the rectangular shape textiles of 30 mm × 16 mm for the test.

The textiles’ ability to attenuate the energy of the incident electromagnetic waves is evaluated as *EMI SE*. As electromagnetic radiation into electromagnetic interference shielding equipment, the absorption (*A*), reflection (*R*), and transmission (*T*) should add up to 1, exhibiting the shielding phenomenon, that is,

3 $$A{{\mbox{+}}}R{{\mbox{+}}}T{{\mbox{=}}}1$$

And the corresponding reflection (*R*) and transmission (*T*) coefficients were directly obtained from the network analyzer in the form of scattering parameters (*S*_22_, *S*_11_, *S*_21_, and *S*_12_). Therefore, the coefficients of *R* and *T* were calculated as follows:

4 $$R{{\mbox{=}}}{{{\mbox{|}}}{S}_{22}{{\mbox{|}}}}^{2}{{\mbox{=}}}{{{\mbox{|}}}{S}_{11}{{\mbox{|}}}}^{2}$$

5 $$T{{\mbox{=}}}{{{\mbox{|}}}{S}_{21}{{\mbox{|}}}}^{2}{{\mbox{=}}}{{{\mbox{|}}}{S}_{12}{{\mbox{|}}}}^{2}$$

As a result, the shielding reflection (*SE*_R_) and absorption (*SE*_A_) can be estimated according to the coefficients of *R* and *T* follows:

6 $${{SE}}_{{{{{\mathrm{R}}}}}}{{\mbox{=}}}10{\log }\left(\frac{1}{1{{\mbox{-}}}R}\right){{\mbox{=}}}10{\log }\left(\frac{1}{1{{\mbox{-}}}{{{\mbox{|}}}{S}_{11}{{\mbox{|}}}}^{2}}\right)$$

7 $${{SE}}_{{{{{\mathrm{A}}}}}}{{\mbox{=}}}10{\log }\left(\frac{1{{\mbox{-}}}R}{T}\right){{\mbox{=}}}10{\log }\left(\frac{1{{\mbox{-}}}{{{\mbox{|}}}{S}_{11}{{\mbox{|}}}}^{2}}{{{{\mbox{|}}}{S}_{21}{{\mbox{|}}}}^{2}}\right)$$

In addition, the multiple internal reflections can be generally negligible as more than 15 dB. Hence, the total EMI *SE*_T_ is the contributions of reflection (*SE*_R_) and absorption (*SE*_A_), which can be evaluated as follows:

8 $$ SE_{{{{{\mathrm{T}}}}}}=SE_{{{\mathrm{R}}}}+{SE}_{{{{{\mathrm{A}}}}}}$$

The specific shielding effectiveness per thickness (*SSE*_t_) of the textiles can be calculated as follows^13,49^:

9 $${SE}{E}_{{{{{\mathrm{t}}}}}}{{\mbox{=}}}\frac{S{E}_{{{{{\mathrm{T}}}}}}}{\rho t}$$

where *ρ* is the density of the fabricated textiles, *t* is the thickness of the he fabricated textiles.

### Measurement for electrothermal effect

The electrothermal property of single fabricated fibers and textiles was investigated using a DC power supply (MP5020D). The silver paste was used as a contact point to connect the MXene-based fibers and conductive copper wires to accelerate the test. The thermal images and temperature-time curves of the fibers were recorded by an IR thermal imaging instrument of the FLIR A3255SC camera. Moreover, the electrical-thermal behavior of textiles was performed as same as that of single MXene-based fibers.

### Fabrication of the textiles

For the prepared textiles, two methods, including manual weaving and machine weaving, were applied. The manual weaving textile was prepared by knitting MGP-T fibers into the existing textile using embroidery technique. The machine weaving textile was prepared by the plain-weave with MGP-T fibers and cotton yarn using a semi-automatic loom (Y208W, Nantong Sansi Technology Co., LTD) constructed with warp and weft densities of 61 and 33 yarns per inch, respectively.

### Measurement for the textiles

The washing tests procedure was conducted according to the ISO 6330 standard with domestic washing and drying^50^. The prepared textiles with the ultra-compact MGP-T fibers were added to the home washing machine (Midea, MT740S) with the anti-bacterial detergent (Yuri-matic, Yuri Distribution Co. Pte. Ltd) to form a 2.3 kg load, followed by setting the program of a standard washing, rinsing, and spinning cycle for 50 minutes at 40 ºC. After every 10 washing cycles with rinsing and drying, the electrothermal heat effect was tested. Two types of textiles, including manual weaving and machine weaving, were tested for drape behavior, moisture vapor transmission rate (MVTR), and drop absorbency behavior. The drape behavior was tested according to the standard of ISO 9073-9-2008. The test was conducted using image processing technology with textiles of 30 cm in diameter. Then, the MVTR was measured according to the standard of ASTM E96. The three specimens with the same diameter of 68 mm were cut from each fabric sample and mounted on top of the standardized cup. The sample was later sealed with a rubber gasket to prevent vapor escape from the sides. 20 mL of distilled water was placed in each cup. The test was conducted at the temperature of 38 ºC with a relative humidity of 50% for 24 h. Next, the drop absorbency behavior was performed according to the standard of AATCC 22. The spray rating test was carried out using a spray tester with a diameter of 20 cm of the textiles, and ratings were provided according to standard. Three same samples were tested for each test.

## Supplementary Information

Supplementary Information

Description of Additional Supplementary Files

Supplementary Movie 1

Supplementary Movie 2

Supplementary Movie 3

Supplementary Movie 4

Supplementary Movie 5

## Data Availability

The data that support the findings of this study are available from the corresponding author upon request.
