# Supplementary material for: Ultra-compact MXene fibers by continuous and controllable synergy of interfacial interactions and thermal drawing-induced stresses
Source: Nat Commun. 2022 Aug 5;13:4564. doi: 10.1038/s41467-022-32361-6 (PMC9356020; doi:10.1038/s41467-022-32361-6)
Supplement: Supplementary file 1 — Supplementary Information [file 41467_2022_32361_MOESM1_ESM.pdf]

Supplementary Information

Ultra-compact MXene Fibers by Continuous and Controllable Synergy of Interfacial Interactions and Thermal Drawing-induced Stresses

Tianzhu Zhou[1,2], Yangzhe Yu[3], Bing He[1], Zhe Wang[1], Ting Xiong[1], Zhixun Wang[1], Yanting Liu[1], Jiwu Xin[1], Miao Qi[1], Haozhe Zhang[1], Xuhui Zhou[1], Liheng Gao[1], Qunfeng Cheng[2,4*], and Lei Wei[1*]

[1]School of Electrical and Electronic Engineering, Nanyang Technological University, Singapore 639798, Singapore

[2]School of Chemistry, Key Laboratory of Bio-inspired Smart Interfacial Science and Technology of Ministry of Education, Beijing Advanced Innovation Center for Biomedical Engineering, Beihang University, Beijing 100191, China

[3]School of Transportation Science and Engineering, Beihang University, Beijing 100191, China

[4]School of Materials Science and Engineering, Zhengzhou University, Zhengzhou 450001, China

*E-mail: wei.lei@ntu.edu.sg; cheng@buaa.edu.cn

Contents

Supplementary Methods..3
Supplementary Note 1. Characterization of Ti_3AlC_2 and accordion-like MXene....................6
Supplementary Note 2. Characterization of MXene nanosheets..7
Supplementary Note 3. Liquid crystal properties and rheological properties of MXene-glutaraldehyde spinning dispersion..9
Supplementary Note 4. The mechanical properties and conductivities of fabricated fibers with different concentrations and draw ratios...11
Supplementary Note 5. Interpretation of FTIR and XPS spectra...16
Supplementary Note 6. Possible reaction mechanism between MXene nanosheets and glutaraldehyde molecules...17
Supplementary Note 7. WAXS/SAXS patterns and the porosity of fabricated fibers............18
Supplementary Note 8. Interpretation of TGA curves..20
Supplementary Note 9. The mechanical properties and conductivities of fabricated fibers....21
Supplementary Note 10. SEM images of the morphology of fibers......................................23
Supplementary Note 11. In-situ XRD and SAXS characterization..25
Supplementary Note 12. Photograph of MGP-T fibers...26
Supplementary Note 13. Plots of azimuthal angle of MGP-T fibers.....................................27
Supplementary Note 14. SEM and TEM of MGP-T fibers..28
Supplementary Note 15. Mechanical properties of MGP-T fibers...30
Supplementary Note 16. Comparations of the toughness and conductivity............................31
Supplementary Note 17. Finite element analysis of PC hollow tube via thermal drawing......32
Supplementary Note 18. Properties of electromagnetic interference shielding and electrothermal applications...33
Supplementary Note 19. Enlarged photograph of textiles with MGP-T fibers.......................34
Supplementary Note 20. Electrothermal performance retention of a textile...........................36
Supplementary Note 21. Washing durability of a sweater with MGP-T fibers......................37
Supplementary Note 22. In vitro cytotoxicity of MXene-based fibers..................................38
Supplementary Tables...39
Supplementary References...54

Supplementary Methods

1. Preparation of MXene nanosheets dispersion.

The solutions with MXene nanosheets were prepared as follows. 1.8 g Ti_3AlC_2 powders were added to 40 mL solution (9 M HCl) that contained 3.8 g of LiF. Then the solution was stirred at 45 °C for 30 hours. After a complete reaction, the accordion-like MXene product was washed with 9 M HCl solution for three cycles and deionized water for about eight cycles. Each cycle involved 5 minutes of centrifugation at 3500 rpm. Until a supernatant solution of MXene nanosheets reached pH≈7, the resulting sediments were dispersed into 200 mL deionized water with continuous vibration for 13 minutes. Next, the solution was centrifuged at 1500 rpm for 30 minutes, and the obtained supernatant solution was then centrifuged at 3500 rpm for 30 minutes to get sediments. Finally, the sediments were dispersed into deionized water to prepare various concentration solutions of MXene nanosheets.

2. Preparation of MXene fibers via wet-spinning.

MXene ($Ti_3C_2T_x$) nanosheets with 5 wt% weight percentages of glutaraldehyde solution at the concentration from 15 mg mL^{-1} to 50 mg mL^{-1} were used as the spinning solution. The MXene spinning solution was placed in a syringe and extruded through the nozzle with a diameter of 250 μm into the prepared coagulant bath with extruded at a velocity of 300 μL min^{-1} by controlling the speed of the take-up roller with the draw ratio of 2.8. The coagulate bath solution consisted of ammonium chloride (12.5 g), ammonium hydroxide solution (5 mL), and deionized water (1 L). With the optimal concentration of 30 mg mL^{-1} of the spinning solution, the extruded MXene spinning solution in the coagulation solution were drawn with draw ratios of 0.5, 1.0, 1.8, and 2.8 to optimize the draw ratio. Then, the drawn MXene gel fibers were transferred to a washing solution consisting of deionized water to systematic optimization of the concentration and draw ratio.

After systematic optimization of the concentration and draw ratio, MXene ($Ti_3C_2T_x$) nanosheets with different weight percentages of glutaraldehyde solution were used as the spinning solution with the concentration of 30 mg mL^{-1}. The extruded MG fibers in the coagulate bath solution at the draw ratio of 2.8 were transferred to a washing bath of deionized water, PVA solutions, and another washing bath of deionized water. Especially, the MG fibers were prepared via adjusting the weight ratios of MXene nanosheets and glutaraldehyde. Furthermore, the MGP fibers with different weight of PVA was obtained by adjusting the speed

of take-up roller when the fibers in the PVA solution. Pure PVA fibers were prepared by the same wet spinning with the coagulant of acetone.

3. Preparation of ultra-compact MGP-T fibers via thermal drawing.

The PC hollow column was prepared via the wrapped PC on the ceramic rod with a diameter of ~6.35 mm and stored in the vacuum oven at 160 °C for 2 hours. Then the ultra-compact MXene-based fibers were fabricated by the thermal drawing process via placing the preform in a two-zone heating furnace, where the top and bottom zones were heated to 150 °C and 350 °C, respectively. Next, the perform with PC hollow column was fed into the furnace with various draw-down ratios (τ) when the MGP fibers were fed into the top of the hollow column. Finally, the ultra-compact fibers were collected labeled as MGP-T. Here, the draw-down ratio (τ) could be defined as follows:

$$\tau=\sqrt{\frac{v_{\mathrm{d}}}{v_{\mathrm{f}}}} \tag{1}$$

where v_{d} and v_{f} are the drawing and feeding speed, respectively.

4. Measuring the conductivity of fibers.

The electrical conductivity of the fabricated MXene-based fibers was measured using a Keithley 2700 source meter with the two-point probe method. Silver paste was conducted as a contact point to connect the MXene fibers and conductive. The conductivity (ρ) of the measured MXene fibers was calculated using equation (2):

$$\rho=\frac{L}{SR} \tag{2}$$

where L is the length of the MXene fibers, R is the electrical resistance, and S is the cross-section area of each measured fiber from SEM images.

5. Estimation of the orientation order parameters of MXene-based fibers.

Quantification of the WAXS/SAXS patterns was conducted with scattering vector q (defined as q=4π sinθ/λ, where 2θ is the scattering angle) and azimuthal angle φ as coordinates. The orientation of the MXene nanosheets in fibers was quantified by the (002) reflection from the WAXS patterns. The MXene nanosheets orientation and fibers alignment were quantified by converting the orientation distribution into a Hermans order parameter (f), defined as:

$$f=\langle\frac{3}{2}\cos^2\varphi-\frac{1}{2}\rangle \tag{3}$$

where φ is the azimuthal distribution, and the orientation order parameter can be expanded as:

$$f=\int_0^{\pi}\mathrm{I}(\varphi)\left(\frac{3}{2}\cos^2\varphi-\frac{1}{2}\right)\sin(\varphi)\,\mathrm{d}(\varphi) \tag{4}$$

The intensity is normalized according to

$$\int_0^{\pi} \mathrm{I}(\varphi)\,\sin(\varphi)\,\mathrm{d}(\varphi)=1 \quad (5)$$

6. Estimation of the porosity of MXene-based fibers.

The porosity (p) of the fabricated MXene-based fibers were calculated according to the structural parameters via equation (6):

$$p\ (\%)=\left(1-\frac{\rho_{MF}d_{002}}{\rho_{M}d_{M}}\right)\times 100 \quad (6)$$

where ρ_{MF} is the density of MXene-based fibers, ρ_{M} is the Ti_3C_2 density of 5.2 g cm^{-3}, d_{002} is the d-spacing (002) of the MXene-based fibers, and d_{M} is the d-spacing of Ti_3C_2 crystals (1.02 nm).

7. Estimation of the flexibility of MXene-based fibers.

The flexibilty (f) of a fiber is the reciprocal of the fibers' cross-section bending stiffness (k)[S1] followed by equation (7):

$$f=\frac{1}{k} \quad (7)$$

and the fiber bending stiffness k can be estimated as equation (8)[S2]:

$$k=E_s\pi\frac{d^4}{64} \quad (8)$$

where Es is the Young's modulus of the material and d is the diameter of the fiber. However, the d can be calculated as equation (9):

$$d=\sqrt{\frac{4A}{\pi}} \quad (9)$$

where A is the measured cross-section area of the fibers.

Supplementary Note 1. Characterization of Ti_3AlC_2 and accordion-like MXene

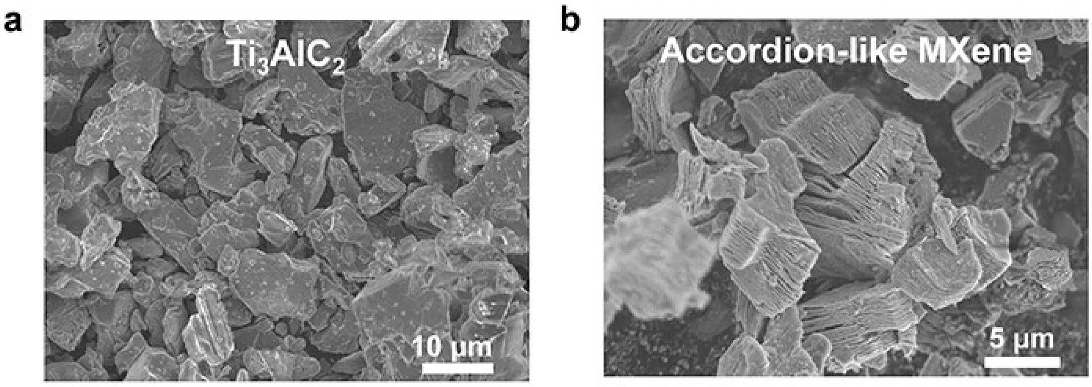

Supplementary Figure 1 | SEM images of **a** Ti_3AlC_2 and **b** accordion-like MXene ($Ti_3C_2T_x$).

Supplementary Note 2. Characterization of MXene nanosheets

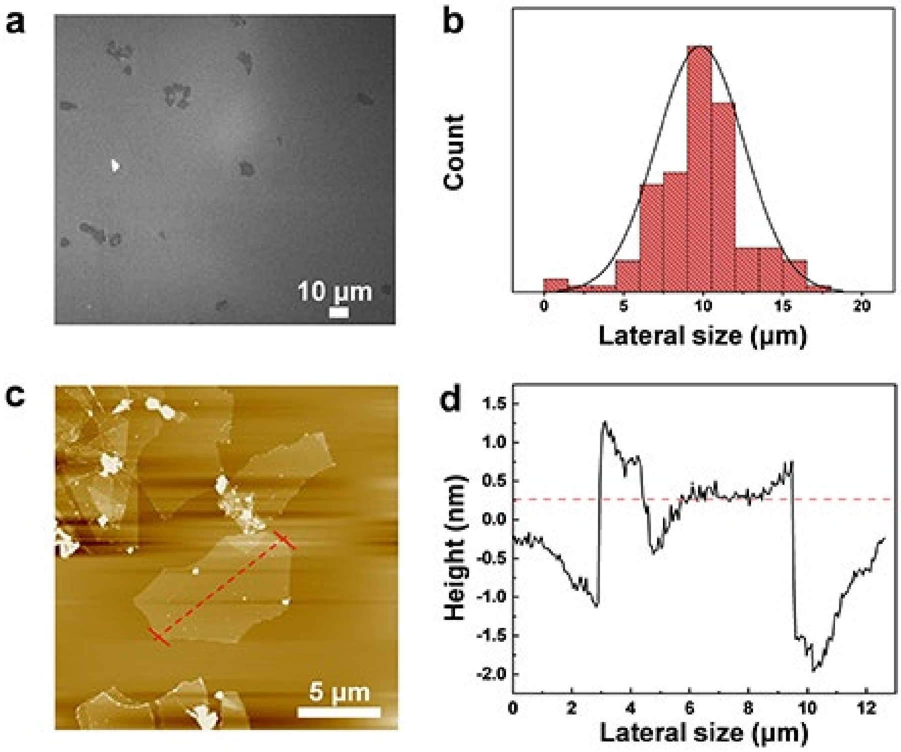

Supplementary Figure 2 | a and **b** SEM image and the size distribution for exfoliated MXene nanosheets with the lateral size of ~10 μm. **c** and **d** AFM images of exfoliated MXene nanosheets with a thickness of ~1.5 nm.

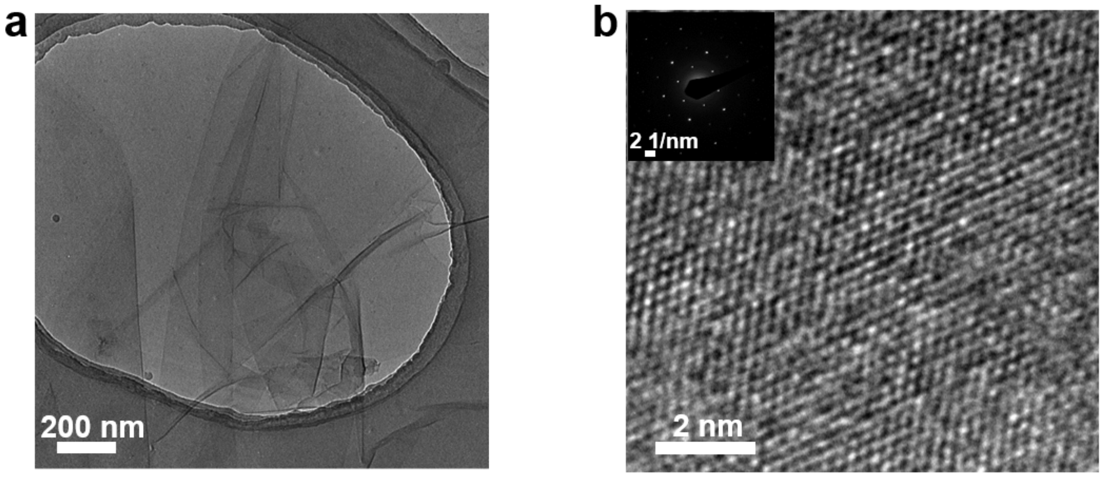

Supplementary Figure 3 | a TEM image and **b** Corresponding HR-TEM image of exfoliated MXene nanosheets. The selected area electron diffraction (SAED) pattern (inset in b) confirms hexagonal single crystals structure without obvious defects.

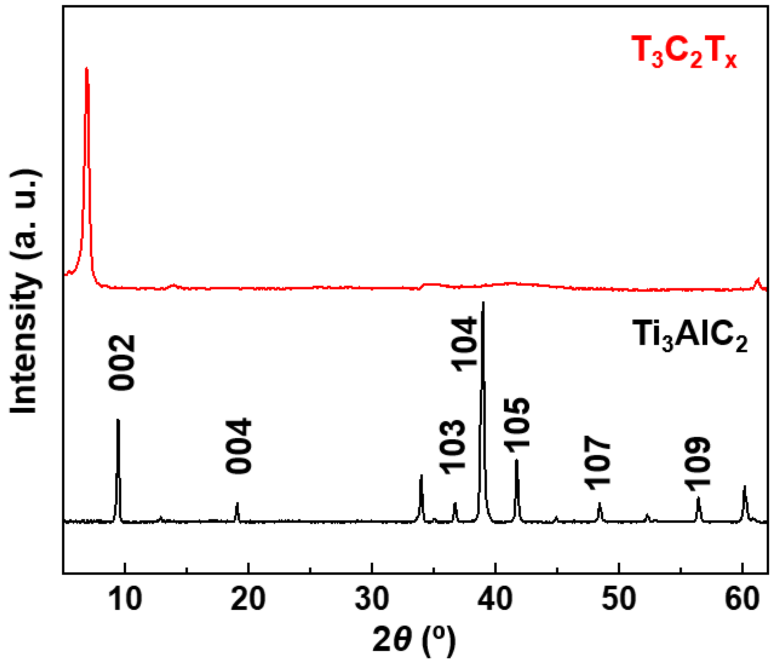

Supplementary Figure 4 | XRD patterns of primitive Ti_3AlC_2 and exfoliated MXene nanosheets. The disappeared (002) and (004) peaks of Ti_3AlC_2 and a new peak at the 2θ of ~6º showed that MXene nanosheets were successfully fabricated from the Ti_3AlC_2.

Supplementary Note 3. Liquid crystal properties and rheological properties of MXene-glutaraldehyde spinning dispersion

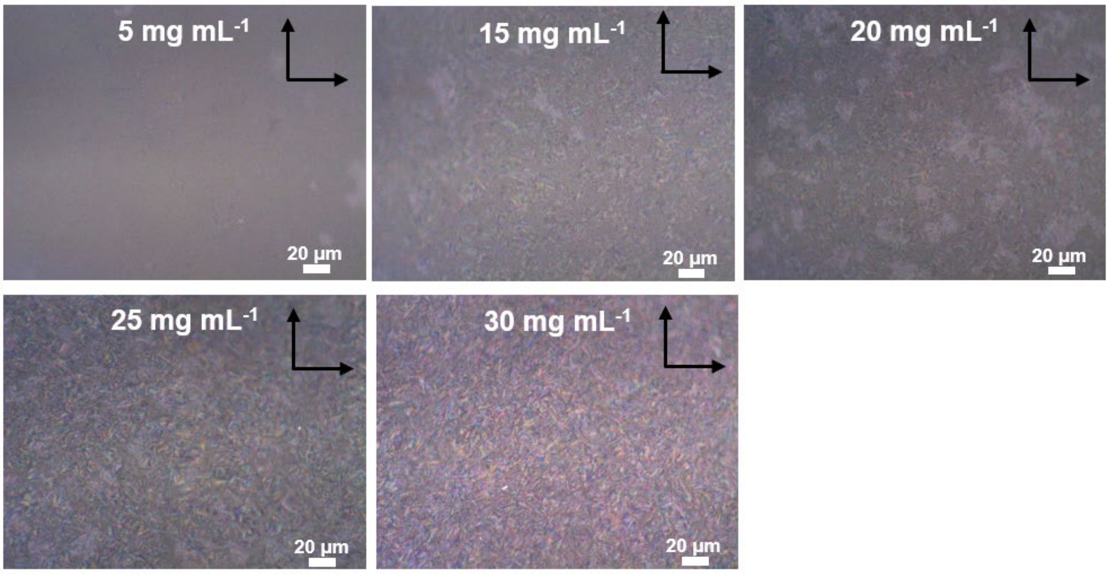

Supplementary Figure 5 | POM images of MXene-glutaraldehyde spinning dispersion with the concentrations from 5 mg mL^{-1} to 30 mg mL^{-1}, which exhibited optical birefringence at the high concentration of 15 mg mL^{-1} and 30 mg mL^{-1}.

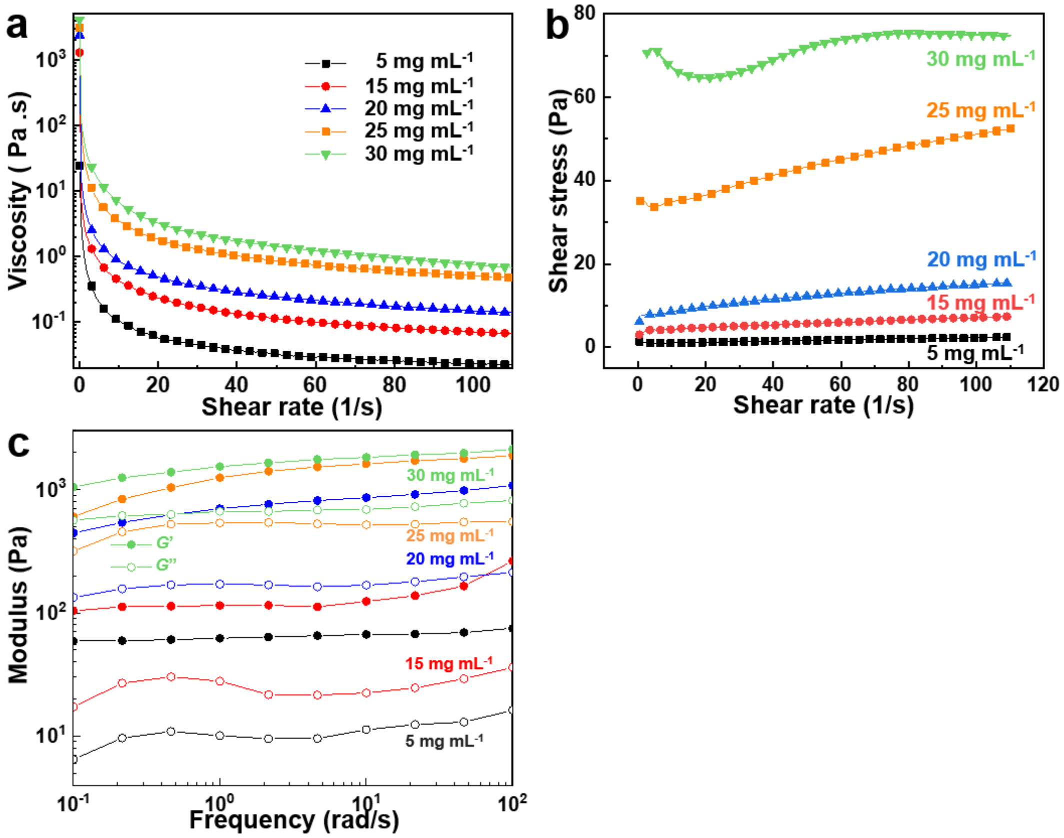

Supplementary Figure 6 | Viscosity (**a**) and Shear stress (**b**) as a function of shear rate (1/s) with different concentrations of MXene-glutaraldehyde spinning dispersion from 5 mg mL^{-1} to 30 mg mL^{-1}. **c** Storage and loss modulus as a function of frequency (rad/s).

Supplementary Note 4. The mechanical properties and conductivities of fabricated fibers with different concentrations and draw ratios

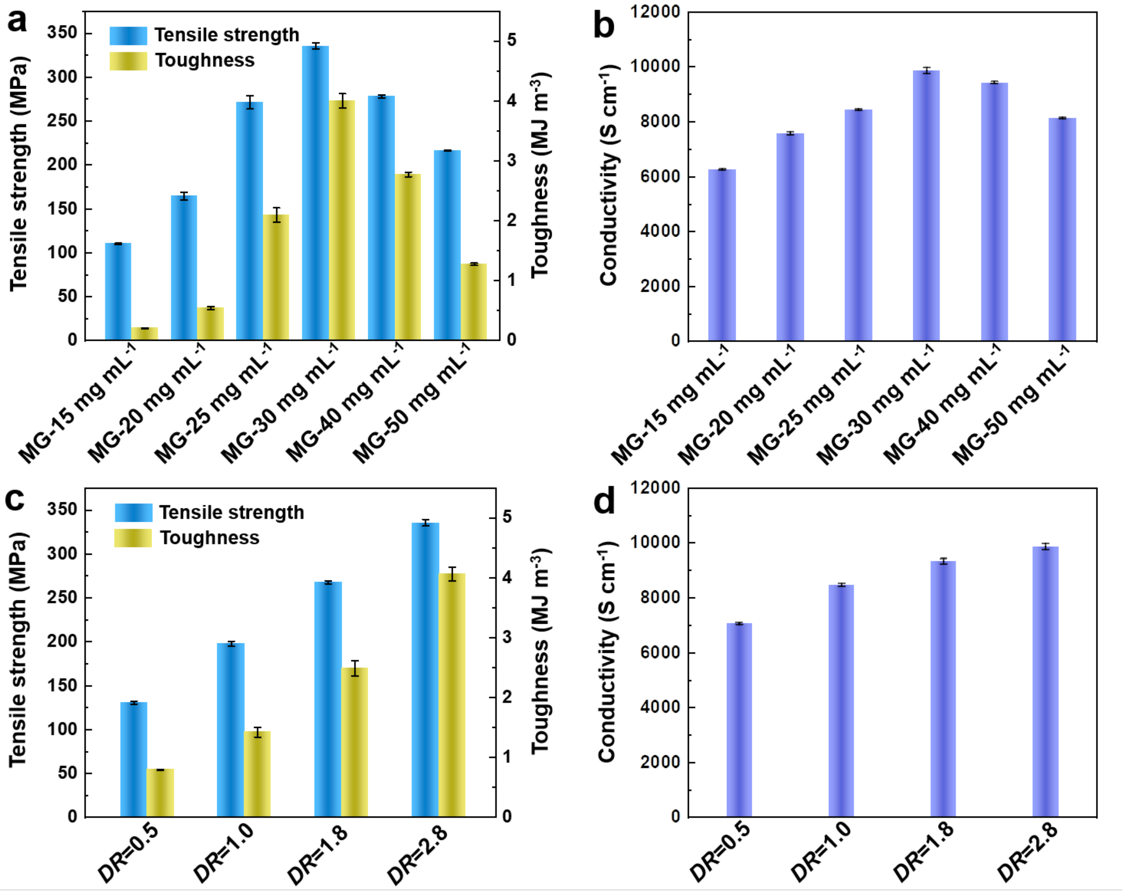

Supplementary Figure 7 | The tensile strengths and toughness (**a**) and conductivities (**b**) of the fabricated MG fibers with various spinning solution concentrations from 15 mg mL^{-1} to 50 mg mL^{-1}. The tensile strengths and toughness (**c**) and conductivities (**d**) of the fabricated MG fibers with various draw ratios from 0.5 to 2.8. All error bars show mean ± standard deviation (SD).

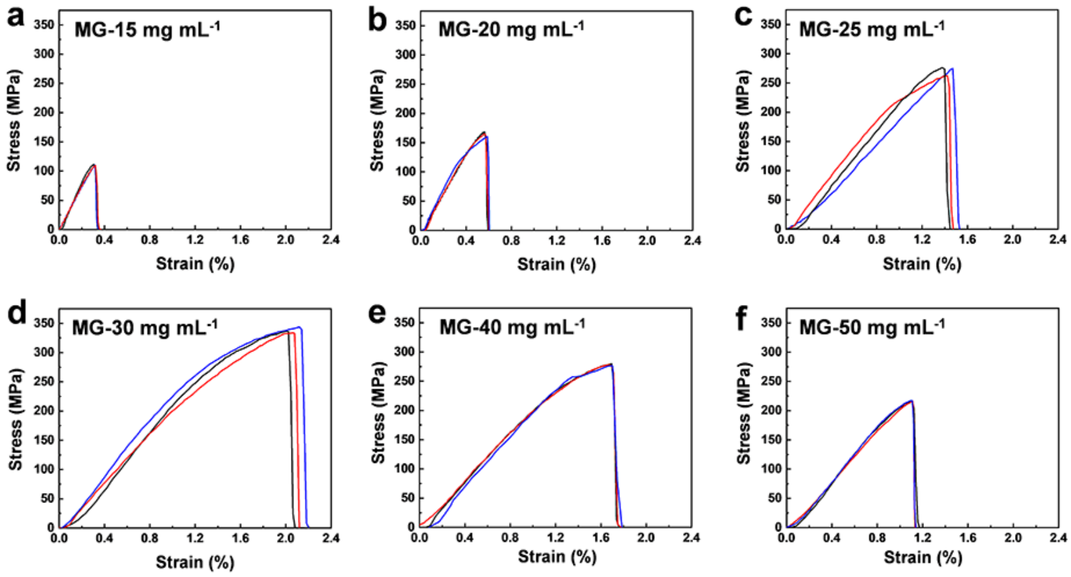

Supplementary Figure 8 | Stress-strain curves for the fabricated MG fibers with various spinning solution concentrations from 15 mg mL^{-1} to 50 mg mL^{-1} of **a** 15 mg mL^{-1}. **b** 20 mg mL^{-1}. **c** 25 mg mL^{-1}. **d** 30 mg mL^{-1}. **e** 40 mg mL^{-1}. **f** 50 mg mL^{-1}.

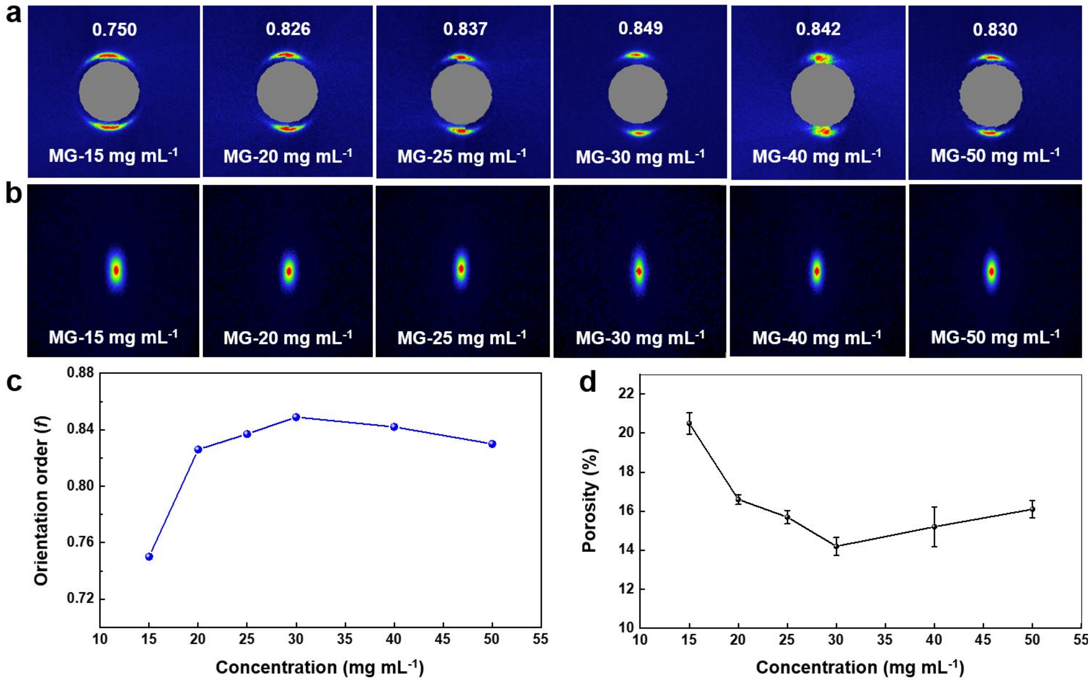

Supplementary Figure 9 | WAXS (**a**) and SAXS (**b**) patterns of fabricated MG fibers with various spinning solution concentrations from 15 mg mL^{-1} to 50 mg mL^{-1}. **c** The orientation order (*f*) of the fabricated MG fibers as a function of various spinning solution concentrations from 15 mg mL^{-1} to 50 mg mL^{-1} according to the WAXS patterns. **d** The porosity of the fabricated MG fibers with various spinning solution concentrations from 15 mg mL^{-1} to 50 mg mL^{-1}. The results show that MG fibers with 30 mg mL^{-1} offer the highest f and lowest porosity. All error bars show mean ± SD.

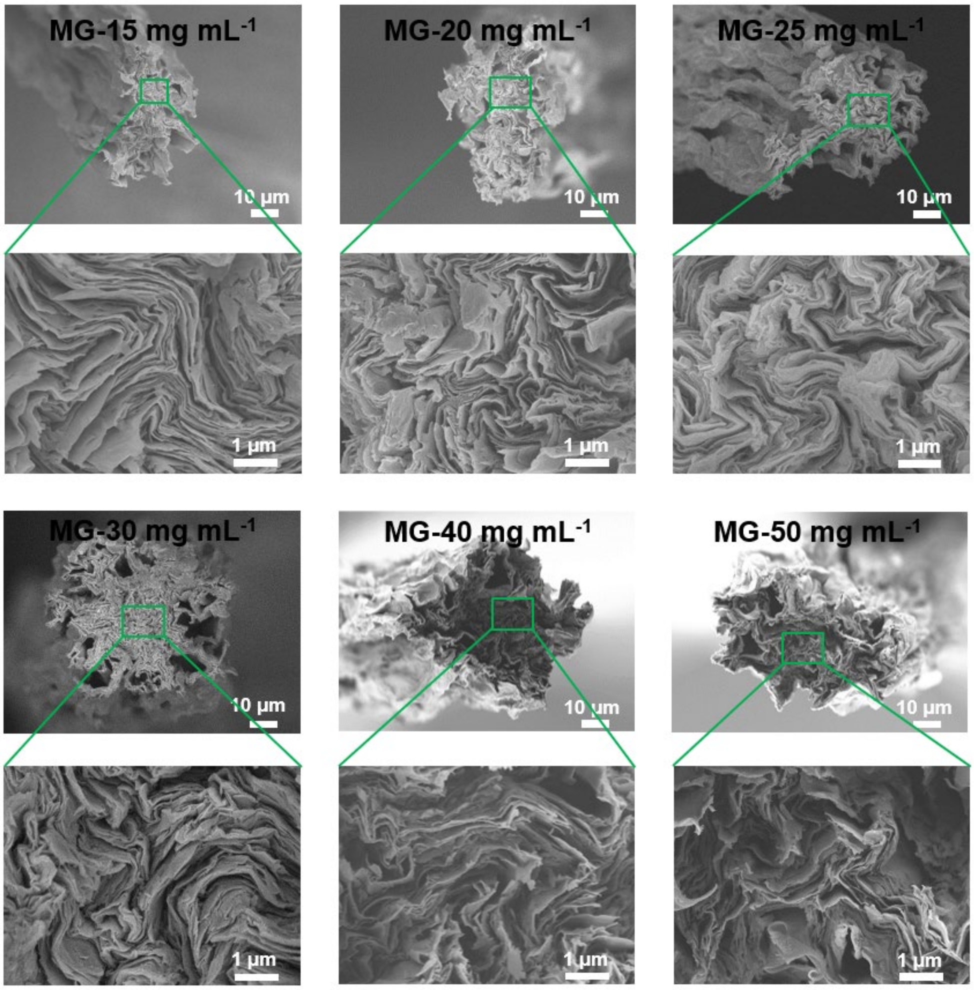

Supplementary Figure 10 | SEM images of the cross-sections of MG fibers with different spinning solution concentration from 15 mg mL^{-1} to 50 mg mL^{-1}.

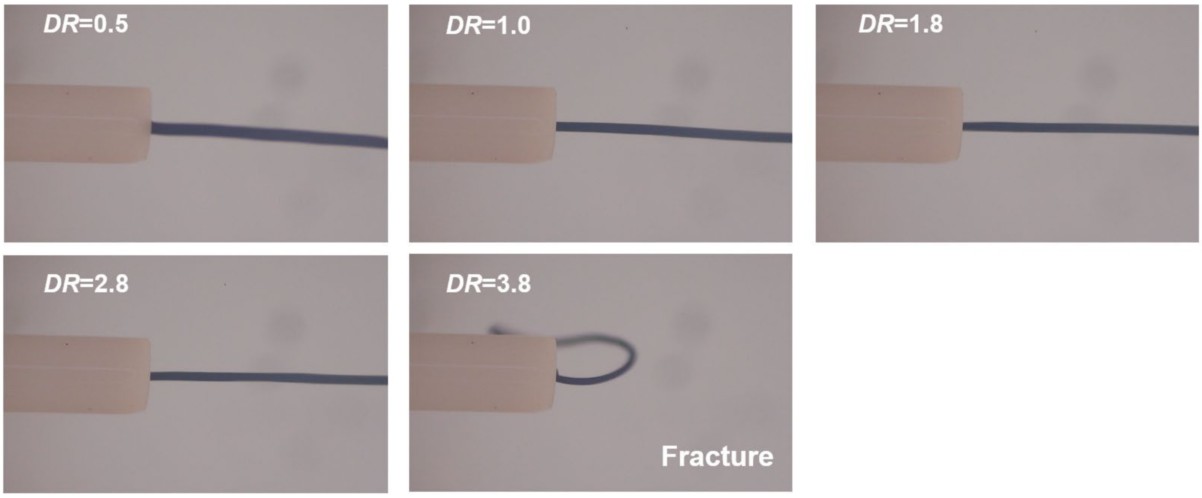

Supplementary Figure 11 | In-situ optical microscopy snapshots of MXene gel fiber at different draw ratios (*DR*) from 0.5 to 3.8 during the wet spinning process.

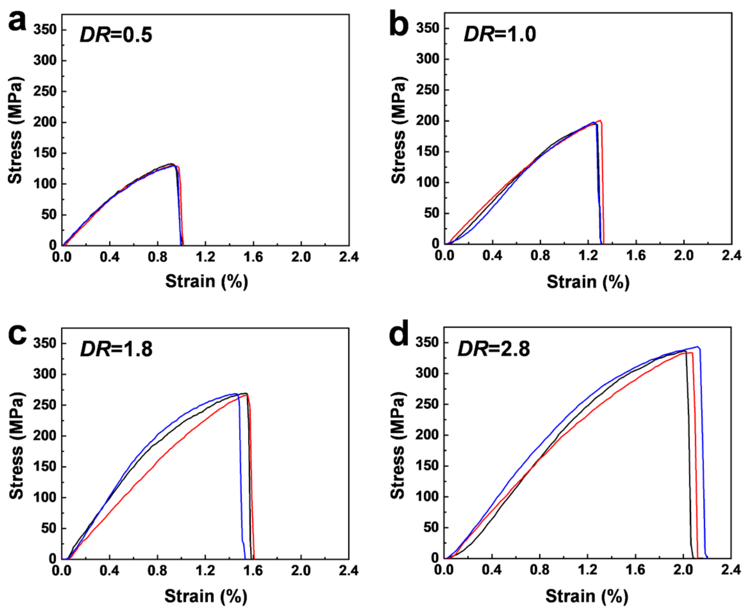

Supplementary Figure 12 | Stress-strain curves for the fabricated MG fibers with various draw ratios (*DR*) of **a** 0.5. **b** 1.0. **c** 1.8. **d** 2.8.

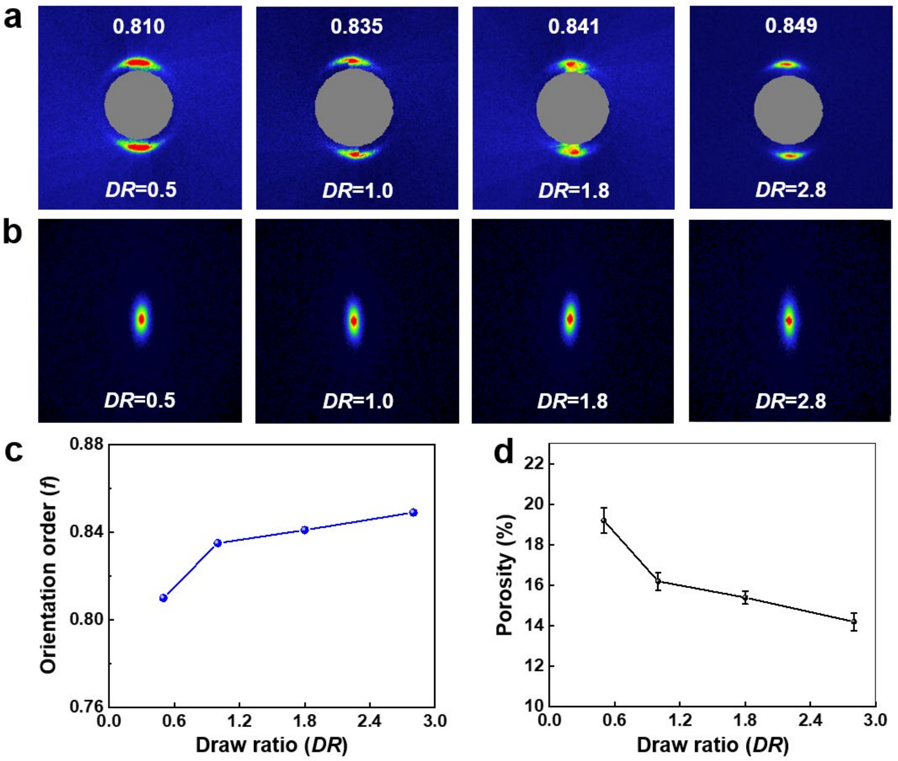

Supplementary Figure 13 | WAXS (**a**) and SAXS (**b**) patterns of fabricated MG fibers with various draw ratios from 0.5 to 2.8. **c** The orientation order (*f*) of fabricated MG fibers as a function of with various draw ratios from 0.5 to 2.8 according to the WAXS patterns. **d** The porosity of fabricated MG fibers with various draw ratios from 0.5 to 2.8. The results show that MG fibers with the *DR* of 2.8 offer the highest f and low porosity. All error bars show mean ± SD.

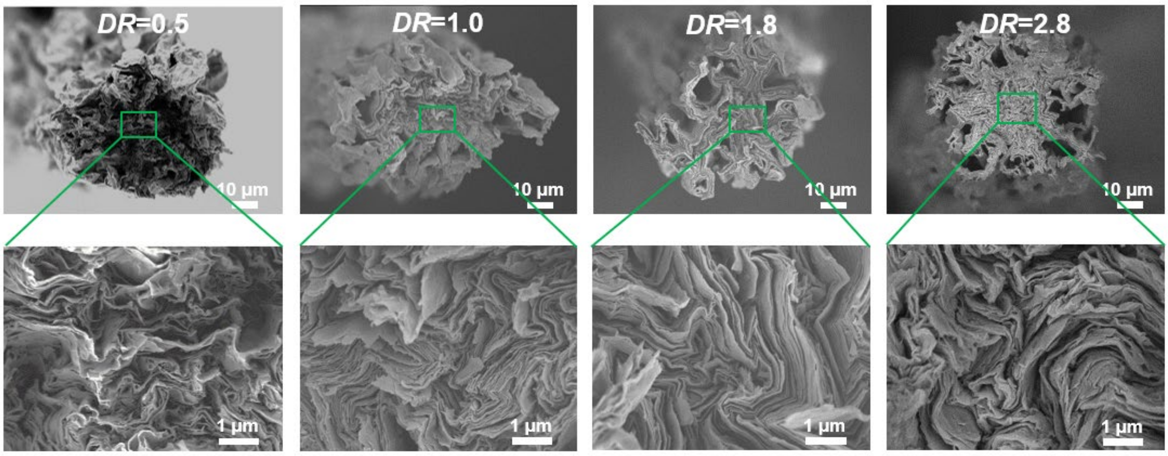

Supplementary Figure 14 | SEM images of the cross-sections of MG fibers with different draw ratios from 0.5 to 2.8.

Supplementary Note 5. Interpretation of FTIR and XPS spectra

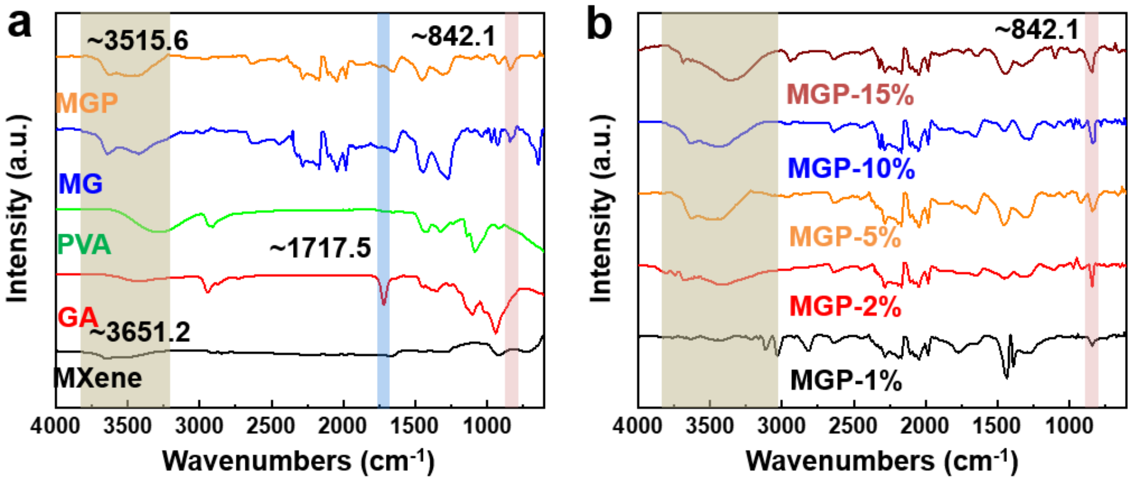

Supplementary Figure 15 | FTIR spectra of **a** MXene-based fibers and **b** MGP fibers with different weight ratios of PVA. The new peak at ~842.1 cm^{-1} and the disappeared peak of the aldehyde group at the wavenumber of ~1717.5 cm^{-1} indicates the formation of the Ti-O-C covalent bond between MXene nanosheets and glutaraldehyde molecules.

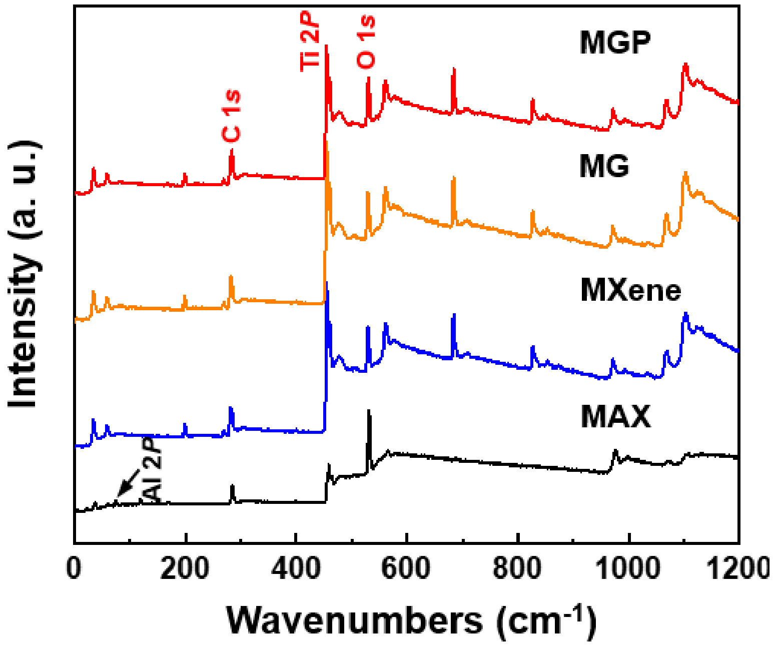

Supplementary Figure 16 | XPS spectra of fabricated fibers. The peaks for Ti and the disappeared Al peak indicate that MXene nanosheets have successfully etched from the primitive MAX (Ti_3AlC_2).

Supplementary Note 6. Possible reaction mechanism between MXene nanosheets and glutaraldehyde molecules

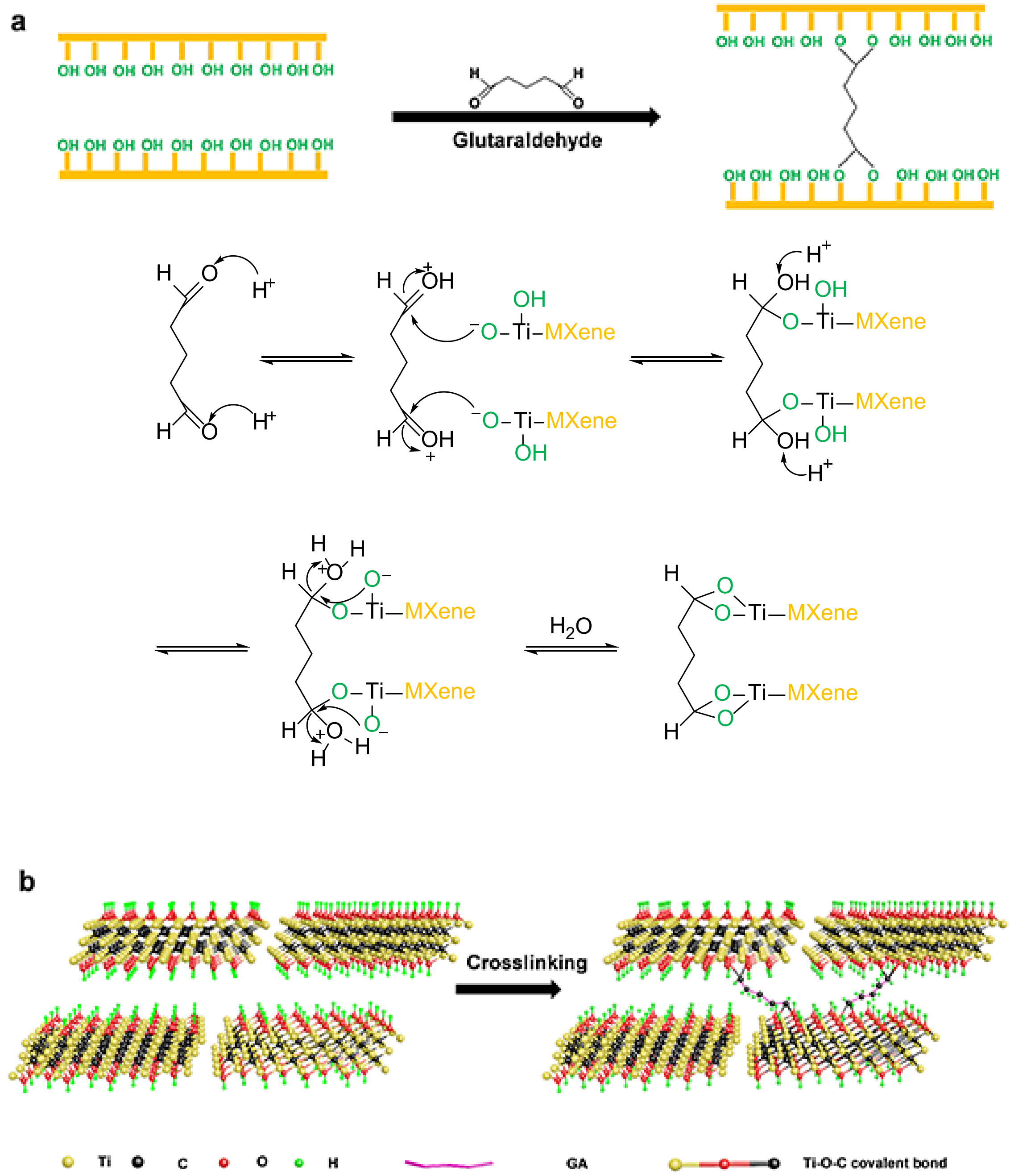

Supplementary Figure 17 | Possible mechanism for the formation of Ti-O-C covalent bond between MXene nanosheets and glutaraldehyde molecules. **a** The aldehyde group (-CHO) of glutaraldehyde molecules reacts with the hydroxyl functional group (OH) of an MXene nanosheet to form Ti-O-C covalent bond via nucleophilic substitution and dehydration reaction. **b** Corresponding structure schematic of formed Ti-O-C bond between MXene nanosheets and glutaraldehyde molecules.

Supplementary Note 7. WAXS/SAXS patterns and the porosity of fabricated fibers

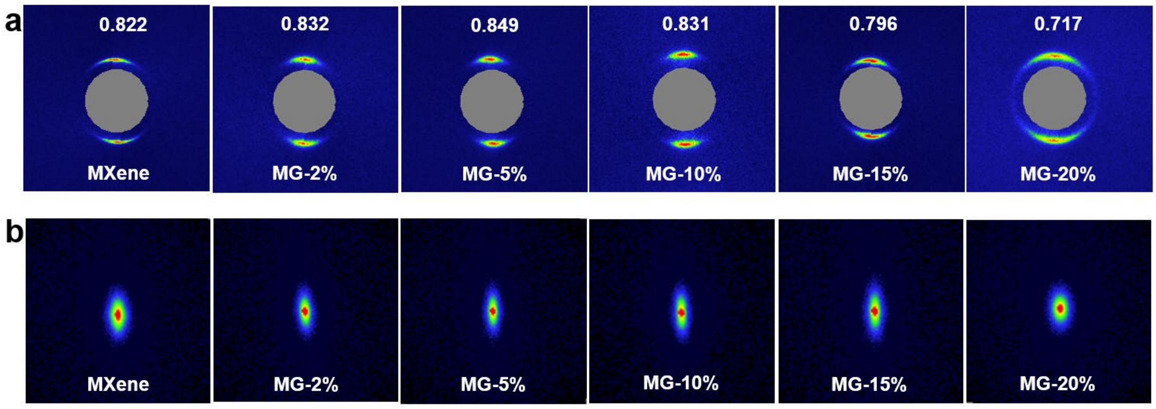

Supplementary Figure 18 | a WAXS patterns of fabricated MG fibers with different weight ratios of glutaraldehyde molecules from 0 wt% to 20 wt%. **b** The corresponding SAXS patterns of fabricated MG fibers.

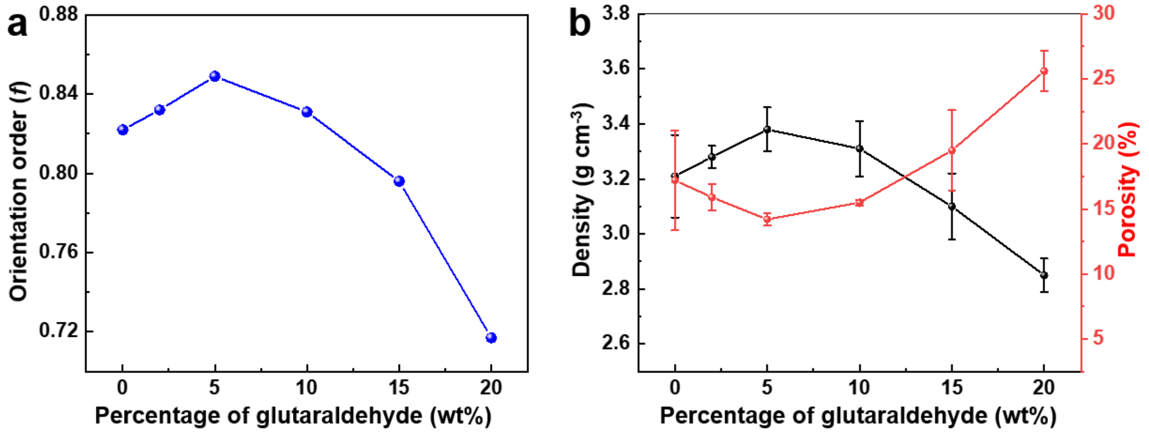

Supplementary Figure 19 | a The orientation order (*f*) of fabricated MG fibers as a function of different weight ratios of glutaraldehyde molecules from 0 wt% to 20 wt% according to the WAXS patterns. **b** Density and porosity of fabricated MG fibers as a function of different weight ratios of glutaraldehyde molecules from 0 wt% to 20 wt%. The results show that MG fibers with 5 wt% glutaraldehyde molecules have achieved the highest *f* and low porosity. All error bars show mean ± SD.

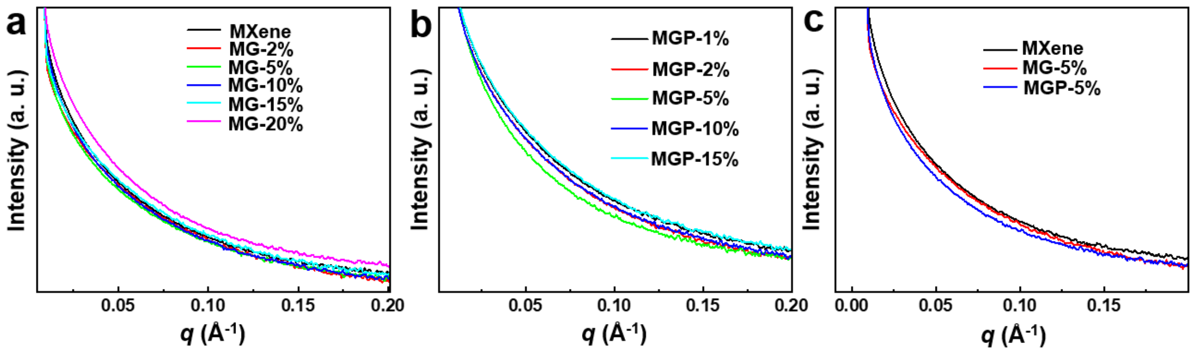

Supplementary Figure 20 | a The intensities curves of the fabricated MG fibers with different weight ratios of glutaraldehyde molecules from 0 wt% to 20 wt%. **b** The intensities curves of the fabricated MGP fibers with different weight percentages of PVA from 1 wt% to 15 wt%. **c** The intensities curves of the fabricated MXene, MG, and MGP fibers.

Supplementary Note 8. Interpretation of TGA curves

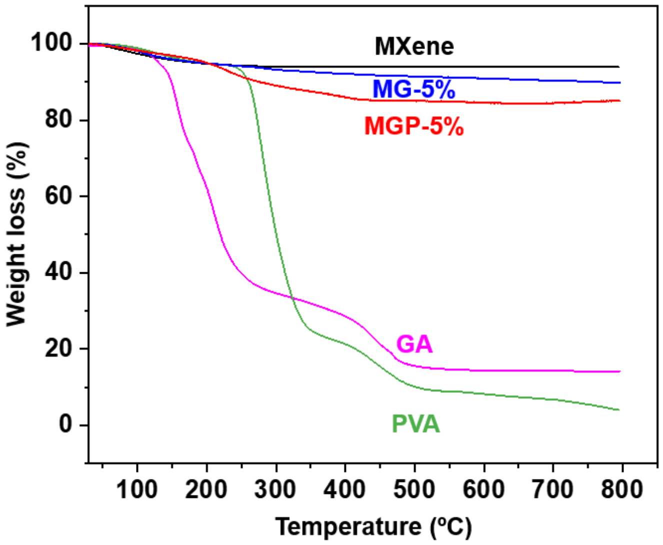

Supplementary Figure 21 | TGA results of PVA, glutaraldehyde (GA), pure MXene fiber, MG-5%, and MGP-5% fibers.

Supplementary Note 9. The mechanical properties and conductivities of fabricated fibers

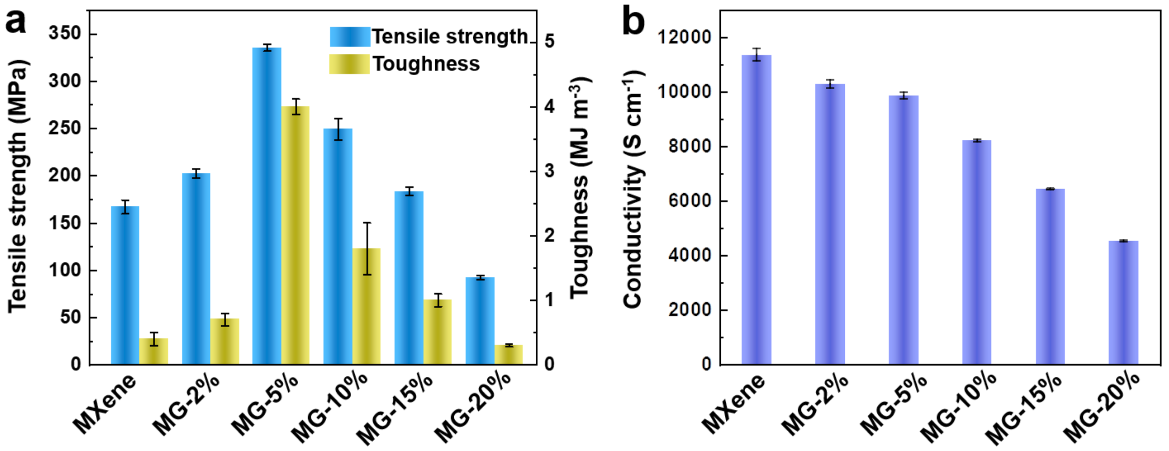

Supplementary Figure 22 | a The tensile strengths and toughness of fabricated MG fibers with various weight percentages of glutaraldehyde molecules from 0 wt% to 20 wt%. **b** The conductivities of fabricated MG fibers. These results show that MG fibers containing 5 wt% glutaraldehyde molecules have maximum tensile strengths, toughness, and conductivity. All error bars show mean ± SD.

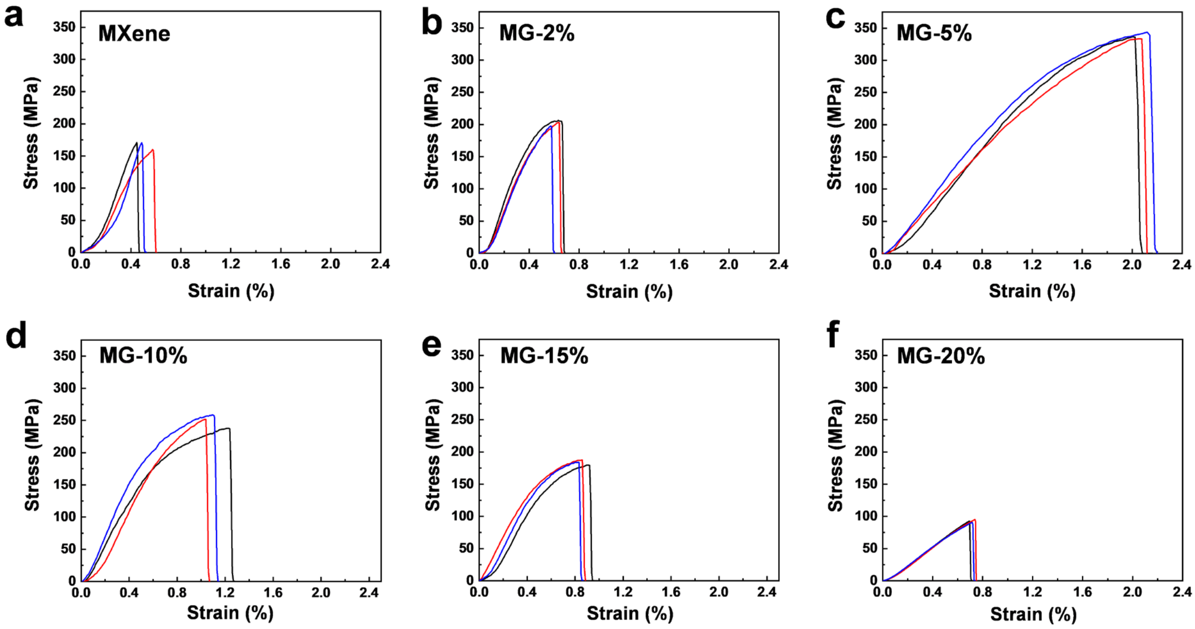

Supplementary Figure 23 | Stress-strain curves for the fabricated MG fibers with various weight percentages of glutaraldehyde molecules of **a** pure MXene. **b** MG-2%. **c** MG-5%. **d** MG-10%. **e** MG-15%. **f** MG-20%.

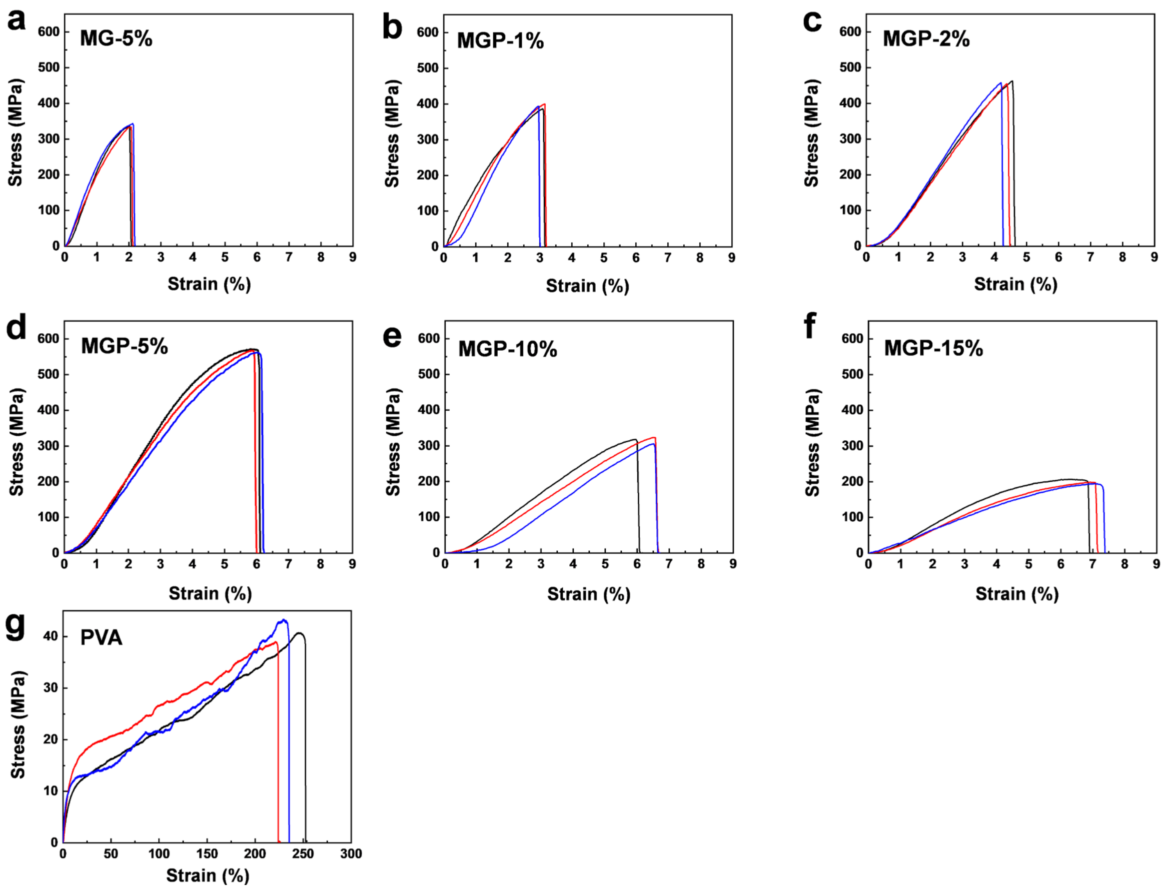

Supplementary Figure 24 | Stress-strain curves for the fabricated MGP fibers with various weight percentages of PVA of **a** MG-5%. **b** MGP-1%. **c** MGO-2%. **d** MGP-5%. **e** MGP-10%. **f** MGP-15%. **g** PVA.

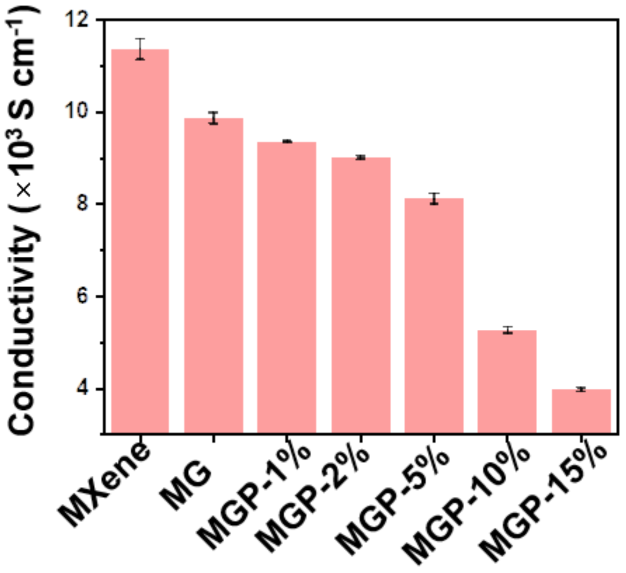

Supplementary Figure 25 | Conductivities of fabricated MGP fibers. These results show that MGP fibers containing 5 wt% PVA molecules have a high conductivity of ~8110.4 S cm^{-1}. All error bars show mean ± SD.

Supplementary Note 10. SEM images of the morphology of fibers

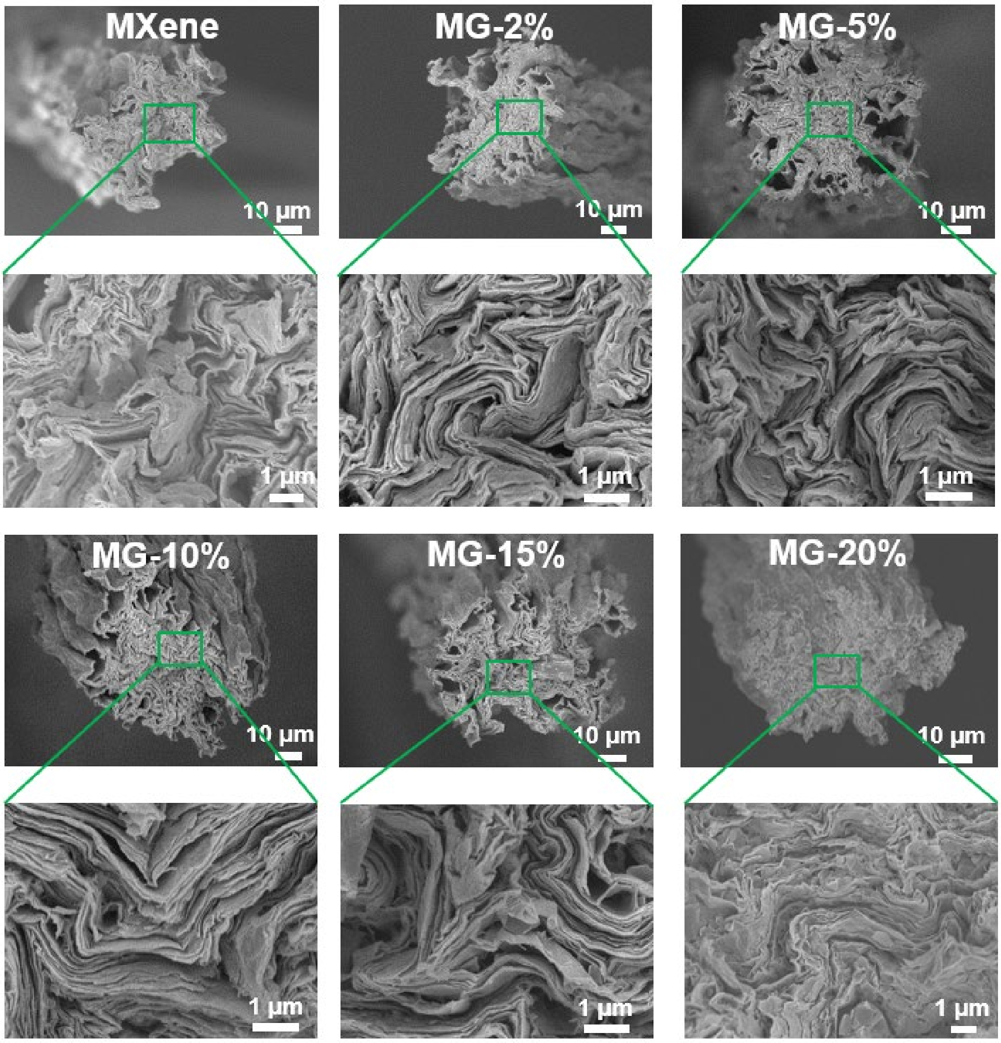

Supplementary Figure 26 | SEM images of the cross-sections of MG fibers with different weight percentages of glutaraldehyde molecules from 0 wt% to 20 wt%.

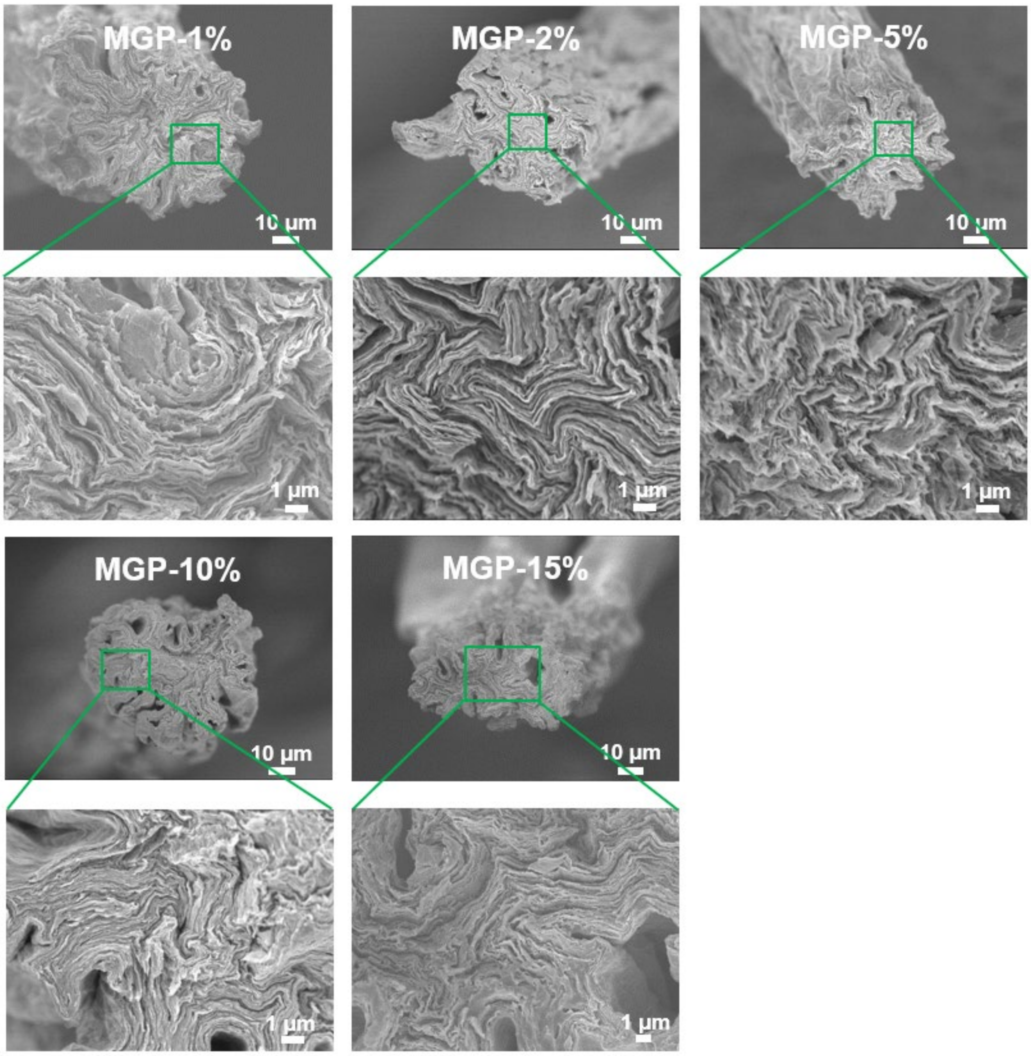

Supplementary Figure 27 | SEM images of the cross-sections of MGP fibers with different weight percentages of PVA from 1 wt% to 15 wt%.

Supplementary Note 11. In-situ XRD and SAXS characterization

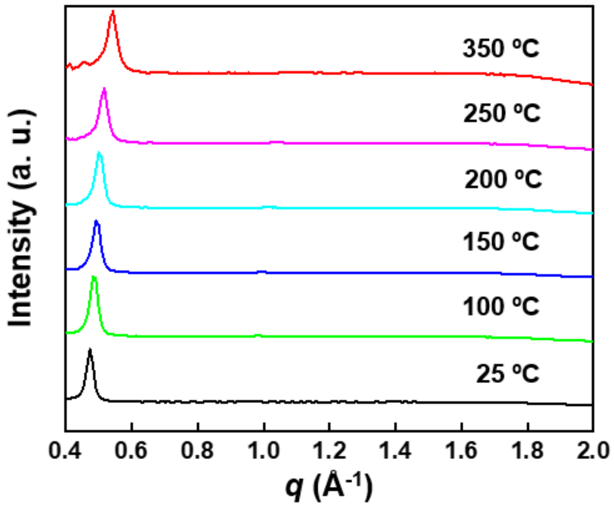

Supplementary Figure 28 | In-situ XRD patterns of fabricated MGP fibers being heated under variable temperature from 25 °C to 350 °C. The results showed the d-spacing between MXene nanosheets was decreased with the increment of heating temperature.

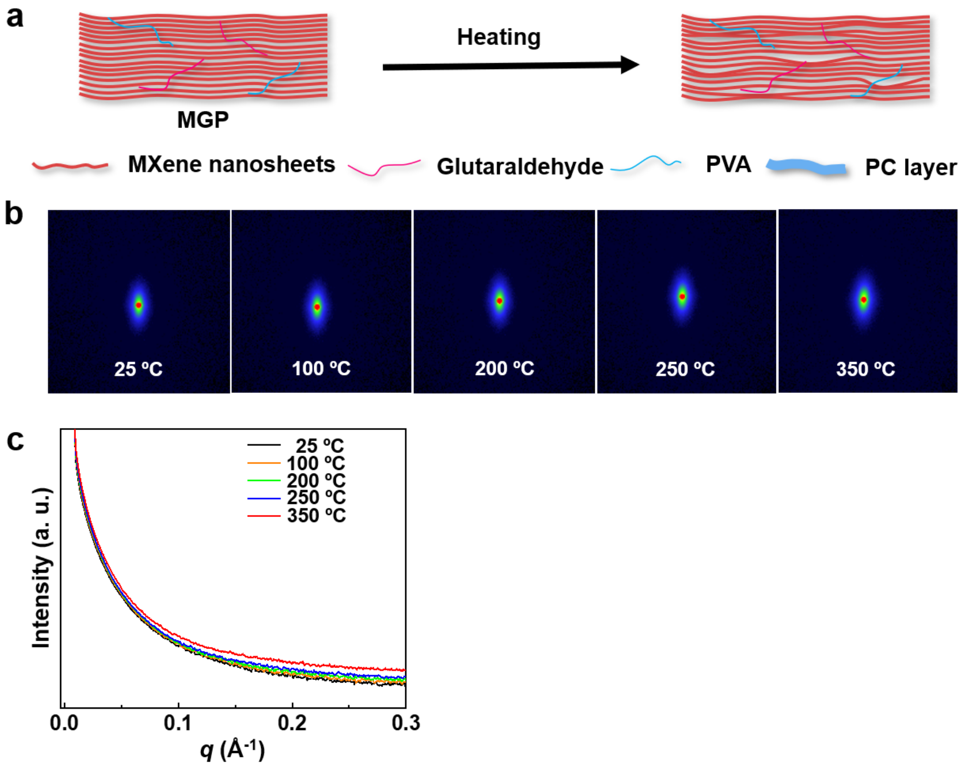

Supplementary Figure 29 | a Schematic diagram of MGP fibers being heated. **b** In-situ SAXS patterns of fabricated MGP-5% fibers being heated under variable temperature from 25 °C to 350 °C. **c** The intensities curves of fabricated fibers under the variable temperature according to the SAXS patterns. The intensities were increasing with the increasing temperature, which indicated more voids and wrinkles of Mxene nanosheets were generated when heating.

Supplementary Note 12. Photograph of MGP-T fibers

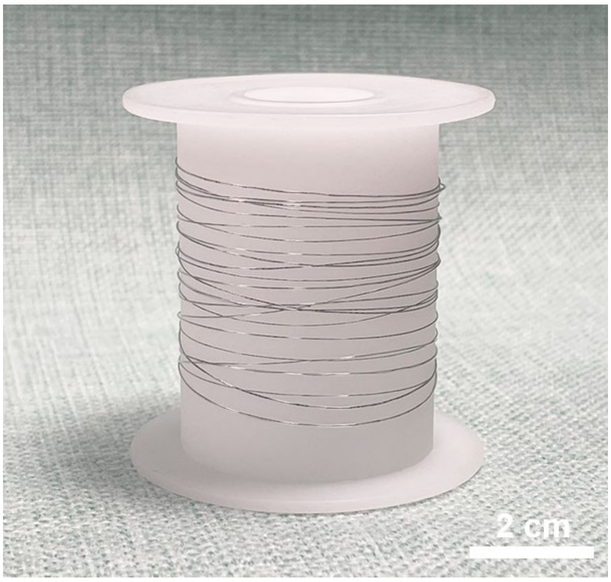

Supplementary Figure 30 | Photograph of MGP-T fibers with several meters long.

Supplementary Note 13. Plots of azimuthal angle of MGP-T fibers

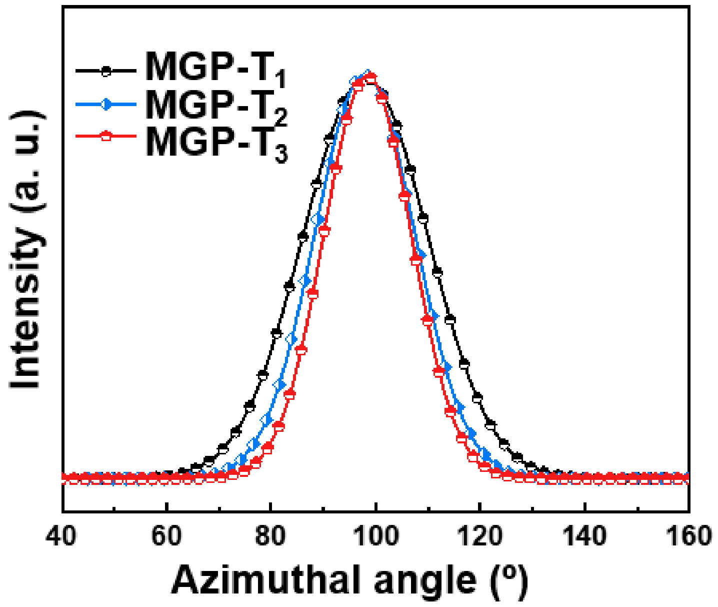

Supplementary Figure 31 | Plots of azimuthal angle MGP-T fibers fabricated by various draw-down ratios according to WAXS patterns.

Supplementary Note 14. SEM and TEM of MGP-T fibers

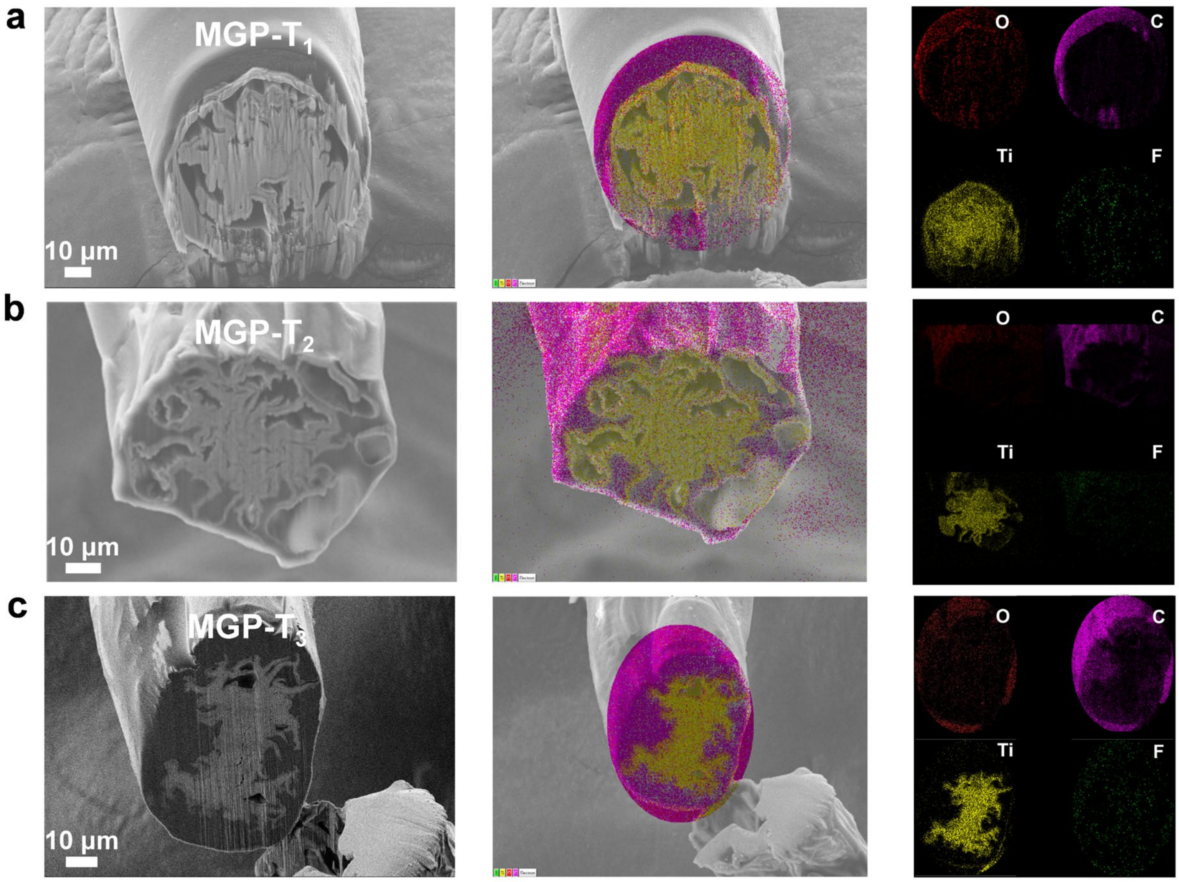

Supplementary Figure 32 | SEM cross-section of MGP-T fibers via different draw-down ratios with the EDS mapping. **a** MGP-T_1 fiber. **b** MGP-T_2 fiber. **c** MGP-T_3 fiber. The results showed that MGP-T fibers get more compact with the increment of the draw-down ratio from MGP-T_1 to MGP-T_3, which significantly reduced the porosity and enhanced the alignment of fibers.

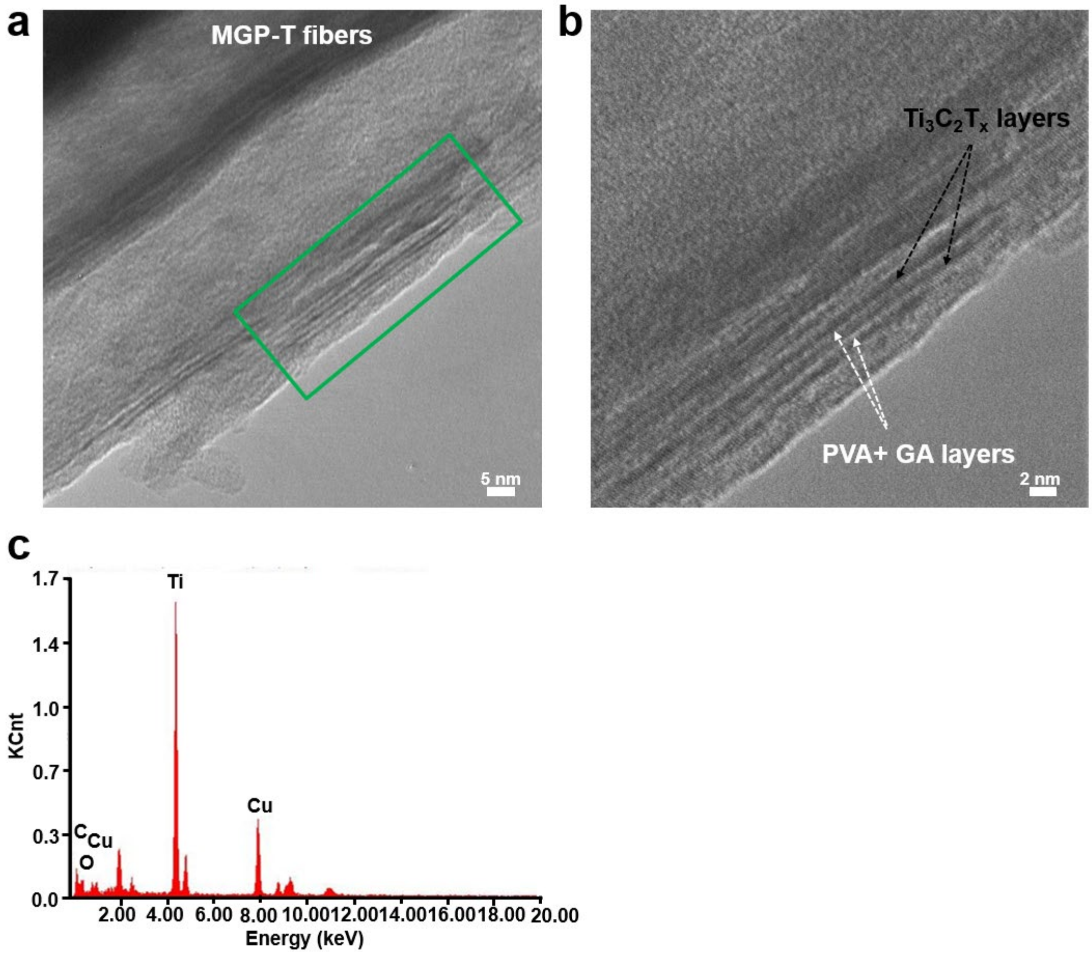

Supplementary Figure 33 | TEM image of MGP-T fiber. a HR-TEM image and **b** Corresponding HR-TEM image of a selected area. The results showed that the PVA/GA polymers (light color) were successfully introduced into the $Ti_3C_2T_x$ layers (dark color) to form MGP-T fibers with high alignment. **c** EDS spectrum of a selected area suggested the existence of O, C, and Ti elements of MXene ($Ti_3C_2T_x$) nanosheets, PVA, and GA polymers.

Supplementary Note 15. Mechanical properties of MGP-T fibers

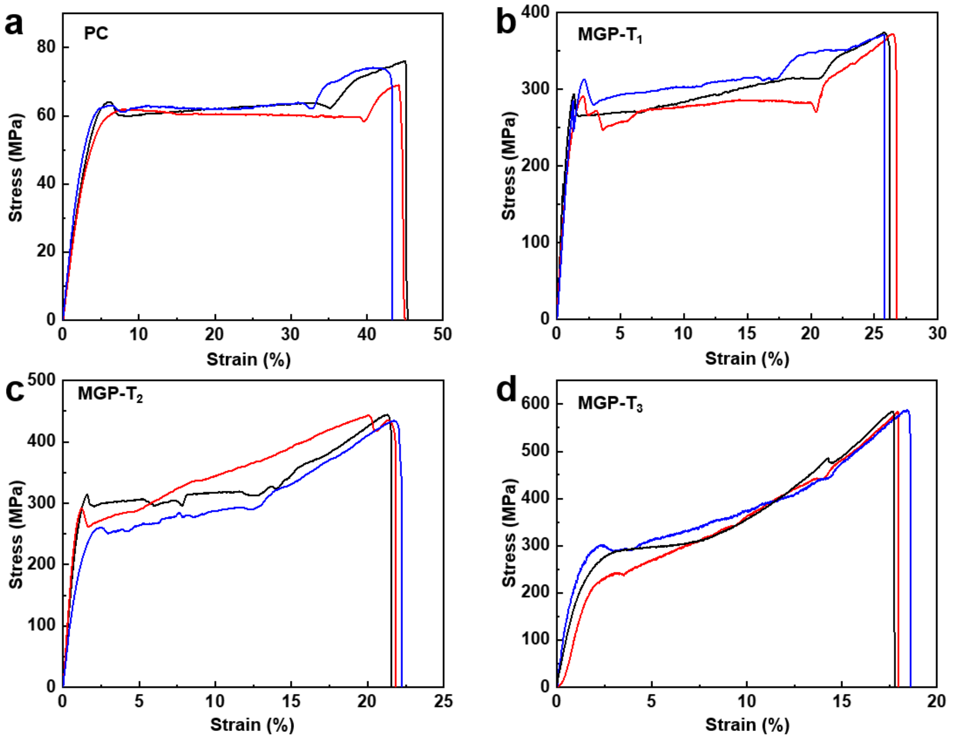

Supplementary Figure 34 | Stress-strain curves for the fabricated MGP-T fibers fabricated by various draw-down ratios of **a** PC. **b** MGP-T$_1$. **c** MGP-T$_2$. **d** MGP-T$_3$.

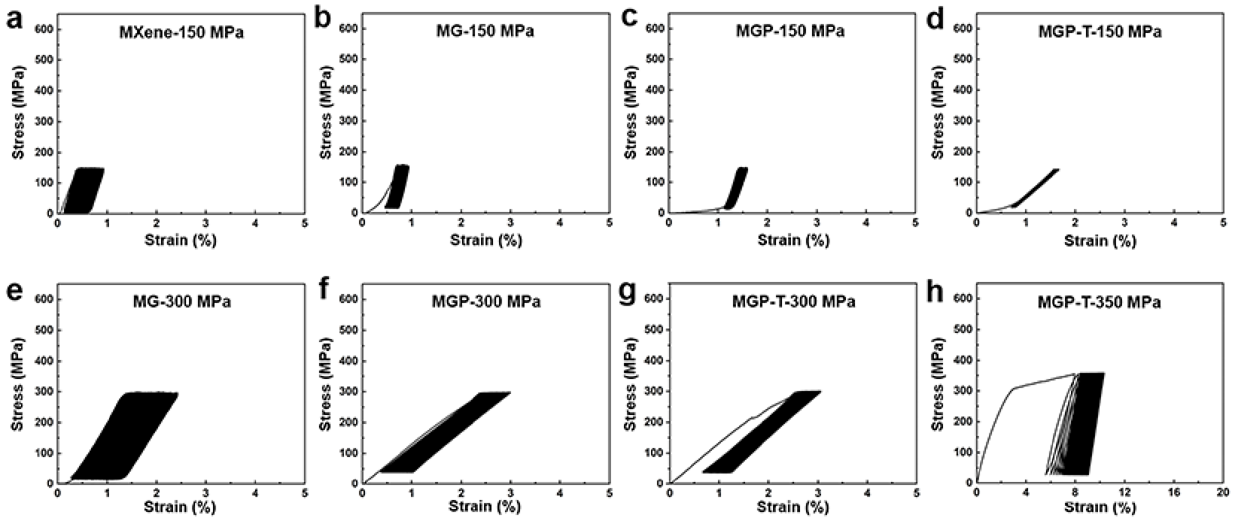

Supplementary Figure 35 | The mechanical stress-strain curves of the pure MXene fiber (**a**), MG fiber (**b**), MGP fiber (**c**), and MGP-T fiber (**d**) at 5000 loading-unloading cycles under the tensile stress of 150 MPa. The mechanical stress-strain curves of the MG fiber (**e**), MGP fiber (**f**), and MGP-T fiber (**g**) at 5000 loading-unloading cycles under the tensile stress of 300 MPa. The mechanical stress-strain curves of MGP-T fiber (**h**) at 5000 loading-unloading cycles under the tensile stress of 350 MPa.

Supplementary Note 16. Comparations of the toughness and conductivity

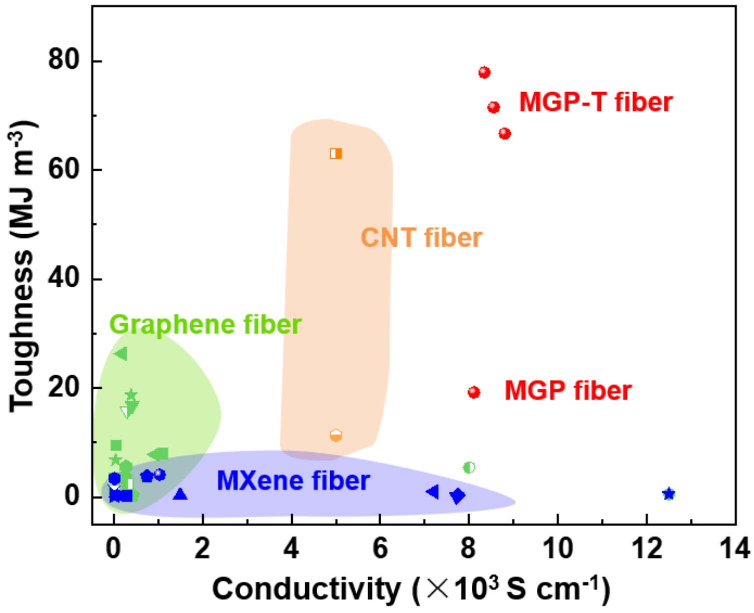

Supplementary Figure 36 | Comparison of the toughness and conductivity of the ultra-compact MXene fibers with that for other reported MXene-based fibers, graphene fibers, and CNT fibers shown in **Supplementary Table 18, 19**. The results showed that the MGP-T with a protective layer had excellent comprehensive properties.

Supplementary Note 17. Finite element analysis of PC hollow tube via thermal drawing

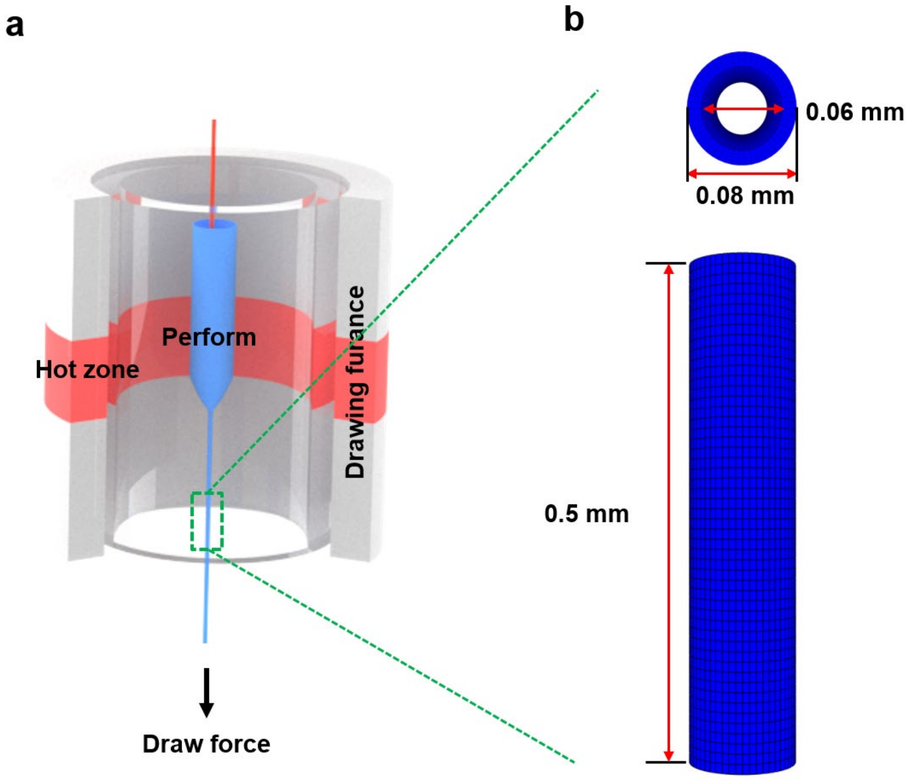

Supplementary Figure 37 | a Schematic diagram of thermal drawing. **b** PC hollow tube model with an inner diameter of 0.06 mm, an outer diameter of 0.08 mm, and 0.50 mm long was constructed to study the mechanical behavior of the PC hollow tube via finite element analysis.

Supplementary Note 18. Properties of electromagnetic interference shielding and electrothermal applications

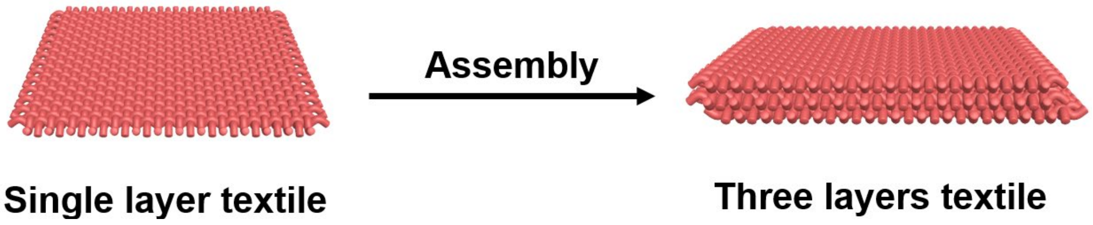

Supplementary Figure 38 | Plain-weaved textiles based on MXene-based fibers for EMI measurements.

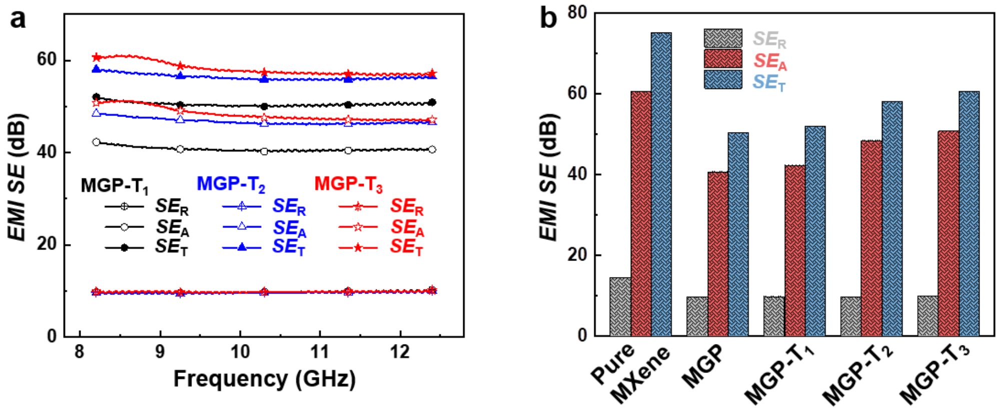

Supplementary Figure 39 | EMI properties of textile with 3 layers based on various MXene-based fibers. EMI SE_R, SE_A, and SE_T of different kinds of textiles based on (**a**) MGP-T fibers at the frequency from 8.2 GHz to 12.4 GHz and (**b**) MXene-based fibers at the frequency of 8.2 GHz.

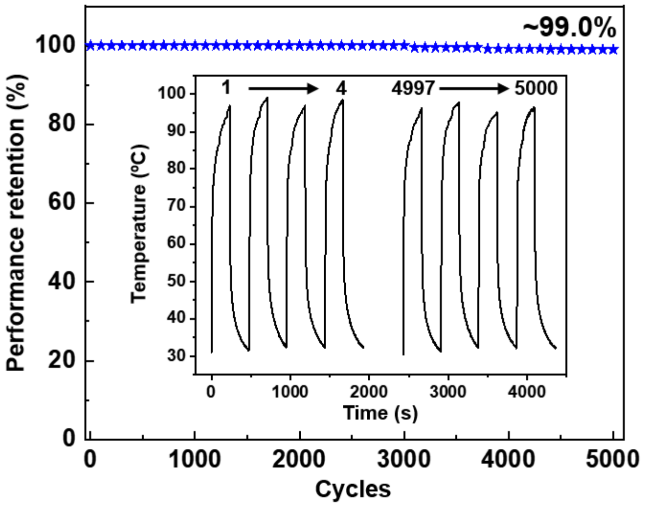

Supplementary Figure 40 | Cycling life of single MGP-T_3 fibers when applied on DC voltage of 6 V. The results showed that the performance retention was ~99% after 5,000 cycles.

Supplementary Note 19. Enlarged photograph of textiles with MGP-T fibers

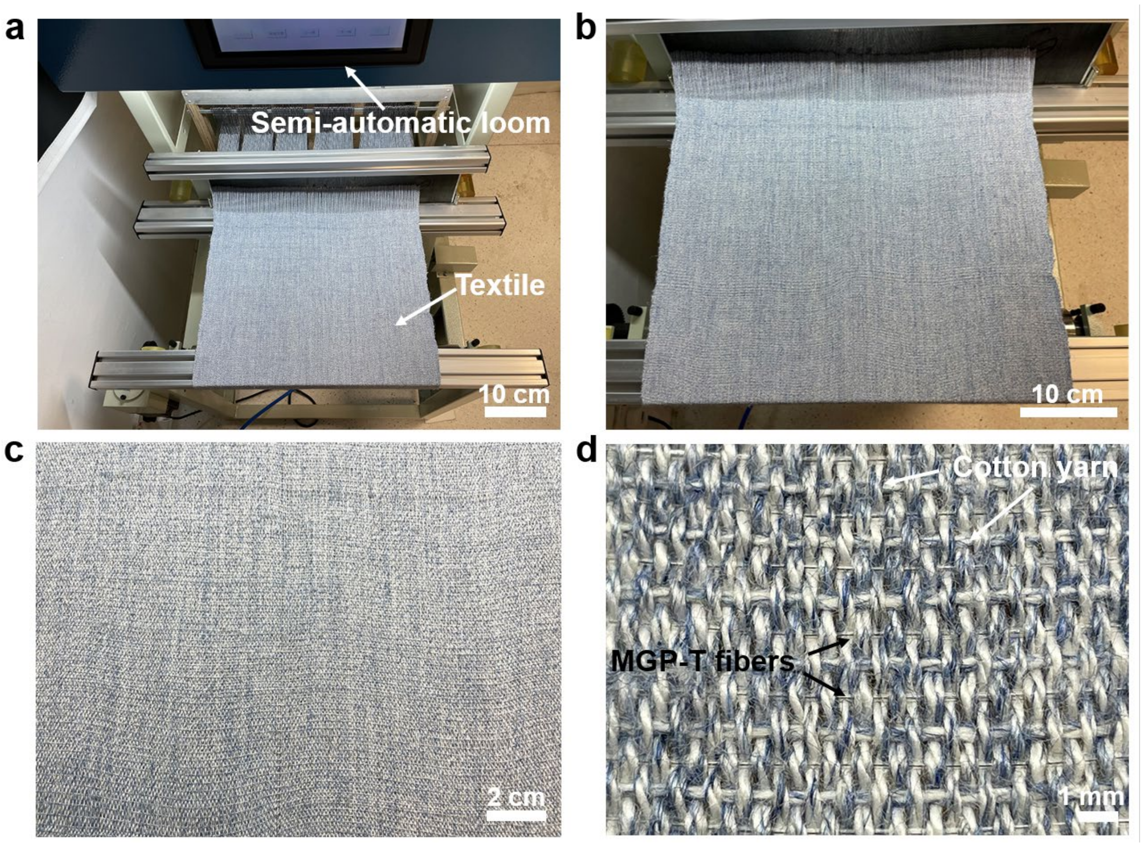

Supplementary Figure 41 | a Photograph of the semi-automatic loom. **b** and **c** Photograph of weaving textiles with the ultra-compact MGP-T fibers. **d** Magnified photograph of the resulting textile with the ultra-compact MGP-T fibers and cotton yarns by machine weaving.

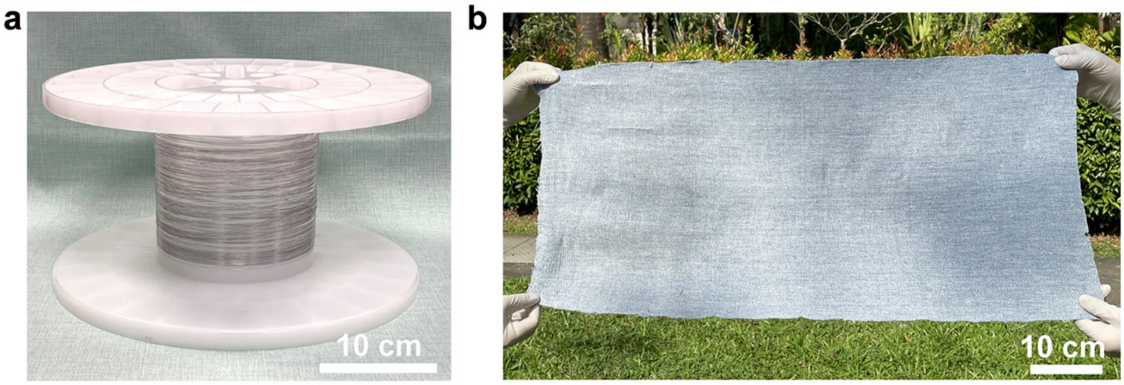

Supplementary Figure 42 | a One hundred and fifty meters long ultra-compact MGP-T fibers for the preparation of the machine weaving textile. **b** A textile (0.8 m by 0.4 m) was prepared via machine weaving with MGP-T fibers.

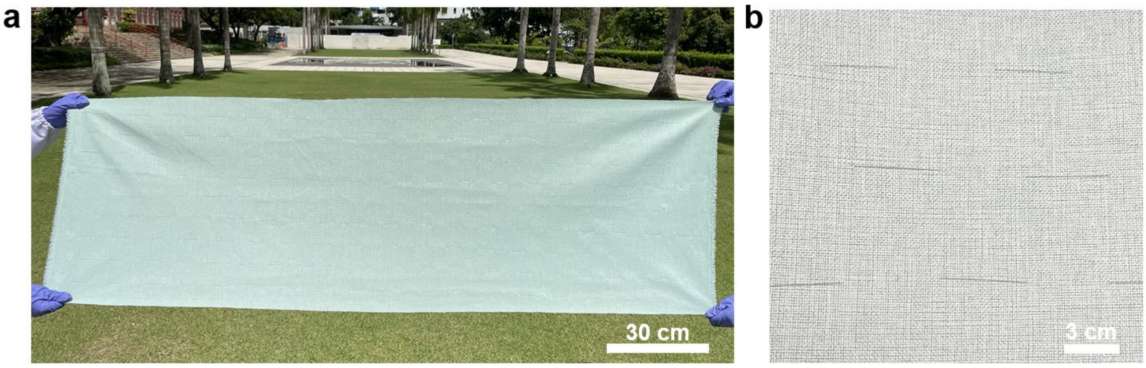

Supplementary Figure 43 | a The 18 meter long ultra-compact MGP-T_3 fibers were woven into a piece of cotton cloth with a length of 2.0 meters and width of 0.6 meters, enabling large-scale applications. **b** The corresponding enlarged photography.

Supplementary Note 20. Electrothermal performance retention of a textile

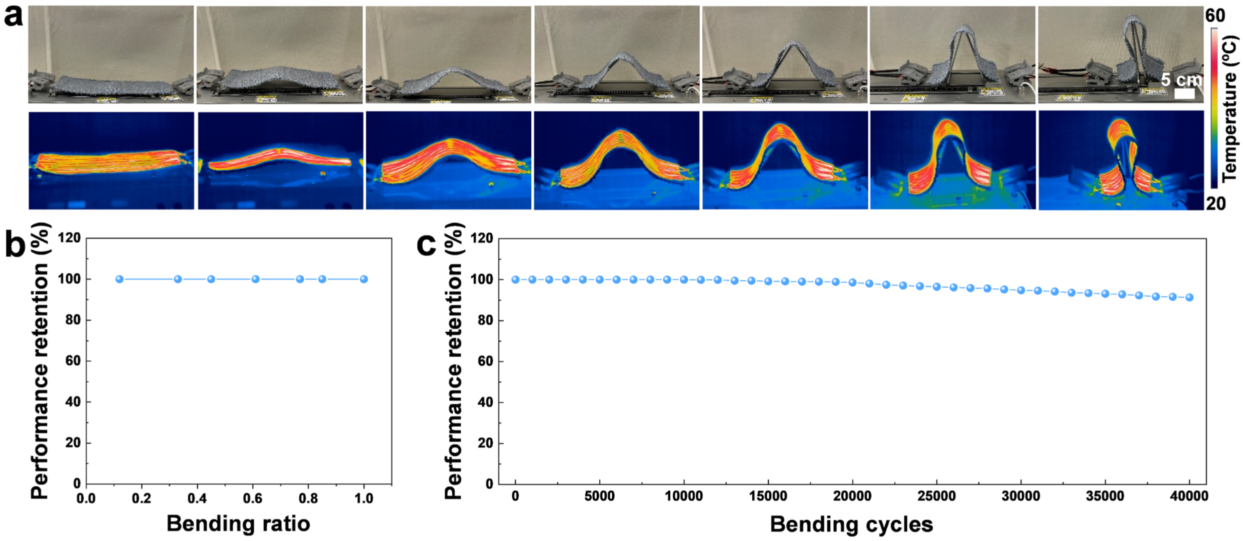

Supplementary Figure 44 | a Photograph of the machine weaving textile with different bending ratios at the applied DC voltage of 4 V. (Bending ratios are the ratios of distance between ends to original length of the textile.) **b** Electrothermal performance retention of the textile suffering from different bending ratios at the applied DC voltage of 4 V. **c** Electrothermal performance retention of the textile after 40,000 bending cycles.

Supplementary Note 21. Washing durability of the textiles with MGP-T fibers

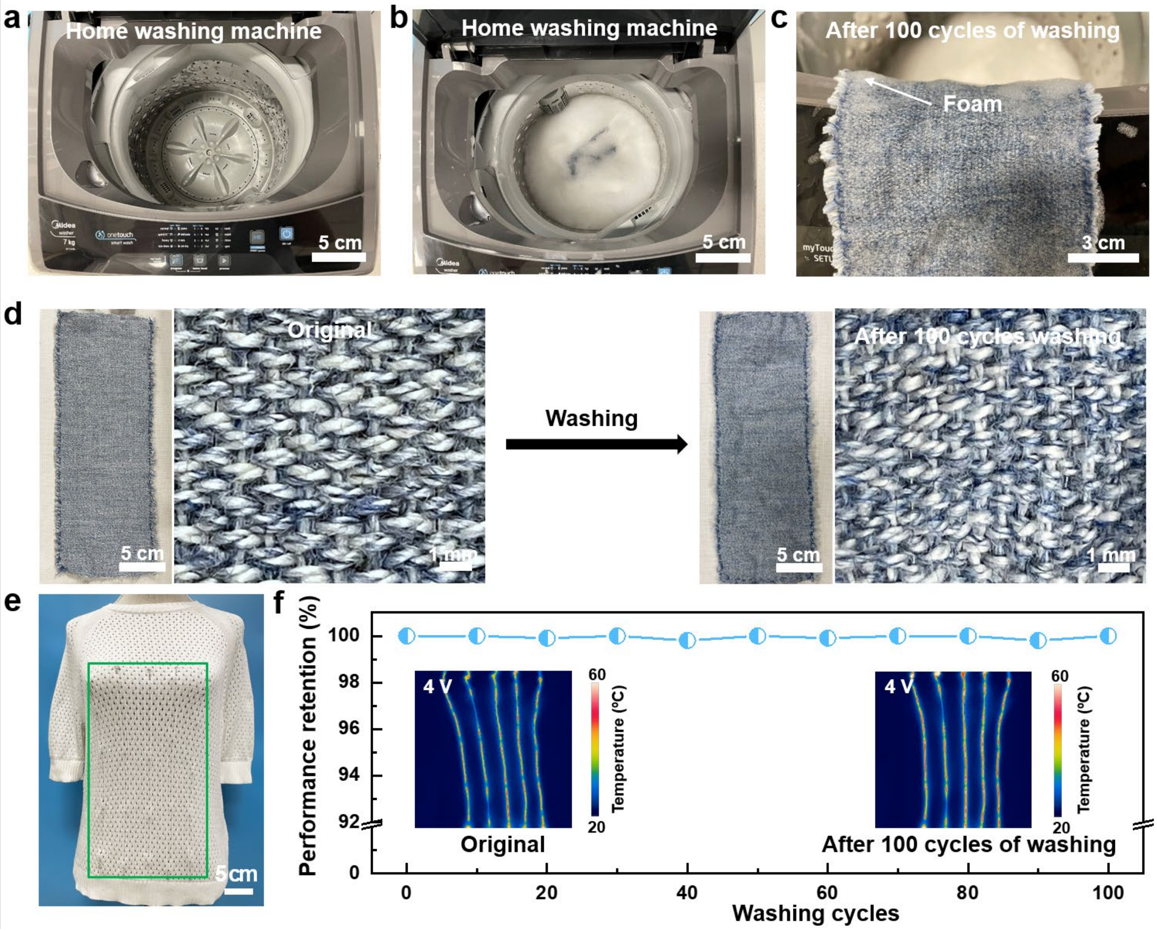

Supplementary Figure 45 | Photograph of the home washing machine (**a**), the washing condition (**b**), and the wet machine weaving textile after 100 cycles of washing (**c**). **d** Photograph of the dry machine weaving textile before and after 100 cycles of washing. **e** Photograph of the white sweater with several MGP-T fibers via manual weaving. **f** Comparison of the performance of the sweater before and after 100 cycles of washing. The results show that the MGP-T fibers in the sweater perform the original state without destruction after 100 cycles of washing.

Supplementary Note 22. In vitro cytotoxicity of MXene-based fibers

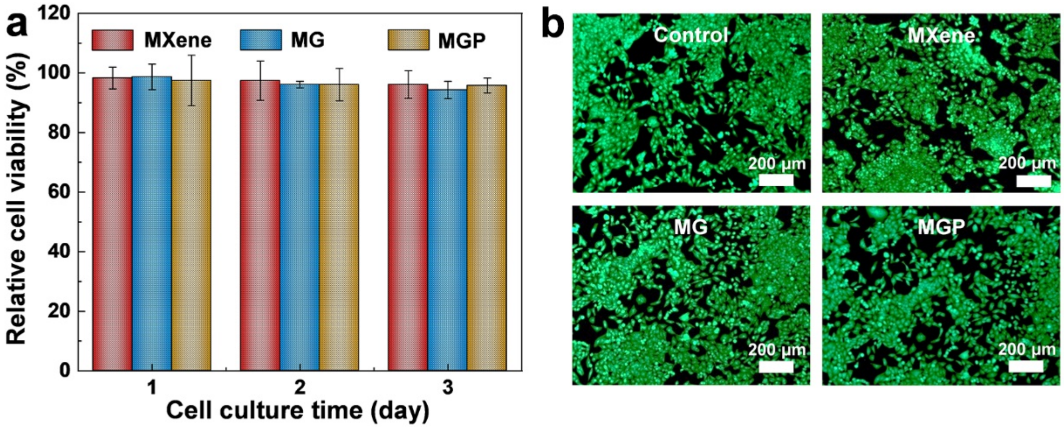

Supplementary Figure 46 | In vitro cytotoxicity results. **a** Relative cell viability for MXene, MG, and MGP fibers after 1, 2, and 3 days of culture. **b** Microscopic images of MC3T3-E1 cells cultured after 3 days in control medium and the extract substrates of pure MXene, MG, and MGP fibers. All error bars show mean ± SD.

The toxicity test process is as follows. For the material extract assay, all materials extract with the concentration of 0.5 mg mL^{-1} were obtained via incubation materials in DMEM media at 37 °C for 24 h. MC3T3-E1 cells suspension was added to 96 well plates and cultured in an incubator with 5% CO_2 at 37 °C for 24 h. After that, the cultured media were removed and the material extracting solution was added, and the solution was cultured for another 24 h. CCK-8 assay was used to determine the cell viability and the cells incubated in culture media without the extracting solution were set as the control group. The relative cell viability (%) is performed as the percentage of the absorbance value relative to the control.

Supplementary Tables

Supplementary Table 1 | The tensile strengths, strain, and toughness of the fabricated MG fibers with various spinning solution concentration from 15 mg mL^{-1} to 50 mg mL^{-1}.

Sample	Stress (MPa) /(CV)	Strain (%) /(CV)	Toughness ($MJ\ m^{-3}$) /(CV)
**MG-15 mg mL^{-1}**	110.2 ± 0.7 (0.01)	0.31 ± 0.01 (0.02)	0.20 ± 0.01 (0.03)
**MG-20 mg mL^{-1}**	164.7 ± 4.2 (0.03)	0.57 ± 0.02 (0.03)	0.54 ± 0.03 (0.05)
**MG-25 mg mL^{-1}**	271.1 ± 7.5 (0.03)	1.42 ± 0.05 (0.03)	2.09 ± 0.12 (0.06)
**MG-30 mg mL^{-1}**	335.6 ± 3.8 (0.01)	2.00 ± 0.06 (0.03)	4.00 ± 0.12 (0.03)
**MG-40 mg mL^{-1}**	278.1 ± 1.7 (0.01)	1.69± 0.01 (0.01)	2.77 ± 0.04 (0.01)
**MG-50 mg mL^{-1}**	216.4 ± 0.6 (0.01)	1.10 ± 0.01 (0.01)	1.27 ± 0.02 (0.02)

Supplementary Table 2 | The electrical conductivity of the fabricated MG fibers with various spinning solution concentration from 15 mg mL^{-1} to 50 mg mL^{-1}.

Sample	Conductivity ($S\ cm^{-1}$)
**MG-15 mg mL^{-1}**	6242.2 ± 24.5
**MG-20 mg mL^{-1}**	7564.4 ± 57.8
**MG-25 mg mL^{-1}**	8632.6 ± 34.2
**MG-30 mg mL^{-1}**	9860.6 ± 117.5
**MG-40 mg mL^{-1}**	9420.3 ± 36.8
**MG-50 mg mL^{-1}**	8123.4 ± 24.2

Supplementary Table 3 | The orientation order (*f*) and porosity of the fabricated MG fibers with various spinning solution concentration from 15 mg mL^{-1} to 50 mg mL^{-1}.

Samples	Orientation order (*f*)	Porosity (%)
**MG-15 mg mL^{-1}**	0.750	20.5 ± 0.54
**MG-20 mg mL^{-1}**	0.826	16.6 ± 0.25
**MG-25 mg mL^{-1}**	0.837	15.7 ± 0.34
**MG-30 mg mL^{-1}**	0.849	14.2 ± 0.45
**MG-40 mg mL^{-1}**	0.842	15.2 ± 1.02
**MG-50 mg mL^{-1}**	0.830	16.1 ± 0.44

Supplementary Table 4 | The tensile strengths, strain, and toughness of the fabricated MG fibers with various draw ratios from 0.5 to 2.8.

Sample	Stress (MPa) /(CV)	Strain (%) /(CV)	Toughness (MJ m^{-3})/(CV)
***DR*=0.5**	130.9 ± 1.8 (0.01)	0.92 ± 0.01 (0.01)	0.79 ± 0.01 (0.01)
***DR*=1.0**	197.8 ± 2.9 (0.01)	1.30 ± 0.03 (0.02)	1.42 ± 0.08 (0.05)
***DR*=1.8**	267.8 ± 1.7 (0.01)	1.52 ± 0.05 (0.03)	2.49 ± 0.13 (0.05)
***DR*=2.8**	335.6 ± 3.8 (0.01)	2.0 ± 0.06 (0.03)	4.00 ± 0.12 (0.03)

Supplementary Table 5 | The electrical conductivity of the fabricated MG fibers with various draw ratios from 0.5 to 2.8.

Sample	Conductivity (S cm^{-1})
***DR*=0.5**	7052.6 ± 36.8
***DR*=1.0**	8452.1 ± 56.4
***DR*=1.8**	9324.5 ± 100.2
***DR*=2.8**	9860.6 ± 117.5

Supplementary Table 6 | The orientation order (*f*) and porosity of the fabricated MG fibers with various draw ratios from 0.5 to 2.8.

Samples	Orientation order (*f*)	Porosity (%)
***DR*=0.5**	0.810	19.2 ± 0.62
***DR*=1.0**	0.835	16.2 ± 0.44
***DR*=1.8**	0.841	15.4 ± 0.32
***DR*=2.8**	0.849	14.2 ± 0.45

Supplementary Table 7 | The atomic percentage of O-Ti-O, C-Ti-OX, O-C, and C-Ti-OH according to the O 1*s* peak in the obtained XPS spectra of MXene, MG, and MGP fibers.

Samples	O-Ti-O (%)	C-Ti-OX (%)	O-C (%)	C-Ti-OH (%)
**MXene**	24.7	33.5	6.2	35.6
**MG**	22.3	29.9	16.4	31.4
**MGP**	20.0	27.7	22.0	30.3

Supplementary Table 8 | The orientation order (*f*) and porosity of the fabricated MG fibers with different weight percentages of glutaraldehyde molecules.

Samples	Orientation order (*f*)	Porosity (%)
**MXene**	0.822	17.2 ± 3.84
**MG-2%**	0.832	15.9 ± 1.00
**MG-5%**	0.849	14.2 ± 0.45
**MG-10%**	0.831	15.5 ± 0.22
**MG-15%**	0.796	19.5 ± 3.09
**MG-20%**	0.717	25.6 ± 1.56

Supplementary Table 9 | The orientation order (*f*) and porosity of the fabricated MGP fibers with different weight percentages of PVA.

Samples	Orientation order (*f*)	Porosity (%)
**MG-5%**	0.849	14.2 ± 0.45
**MGP-1%**	0.861	13.7 ± 2.33
**MGP-2%**	0.863	11.9 ± 3.30
**MGP-5%**	0.872	7.4 ± 1.27
**MGP-10%**	0.865	14.4 ± 3.60
**MGP-15%**	0.850	23.2 ± 3.06

Supplementary Table 10 | The tensile strength and toughness of the fabricated MG fibers with different weight percentages of glutaraldehyde molecules.

Samples	Tensile strengths (MPa)/(CV)	Strain (%)/(CV)	Toughness (MJ m^{-3})/(CV)
**MXene**	167.1 ± 7.2 (0.04)	0.5 ± 0.1 (0.20)	0.4 ± 0.1 (0.25)
**MG-2%**	202.4 ± 4.7 (0.02)	0.6 ± 0.1 (0.17)	0.7 ± 0.1 (0.14)
**MG-5%**	335.6 ± 3.8 (0.01)	2.0 ± 0.06 (0.03)	4.0 ± 0.12 (0.03)
**MG-10%**	249.4 ± 11.4 (0.05)	1.1 ± 0.1 (0.09)	1.8 ± 0.4 (0.22)
**MG-15%**	183.4 ± 4.3 (0.02)	0.9 ± 0.1 (0.11)	1.0 ± 0.1 (0.10)
**MG-20%**	92.6 ± 2.4 (0.03)	0.7 ± 0.02 (0.03)	0.3 ± 0.02 (0.07)

Supplementary Table 11 | The electrical conductivity of the fabricated MG fibers with different weight percentages of glutaraldehyde molecules.

Sample	Electrical conductivity (S cm^{-1})
**MXene**	11,360.4 ± 227.2
**MG-2%**	10,288.3 ± 144.0
**MG-5%**	9,860.6 ± 117.5
**MG-10%**	8,210.5 ± 46.2
**MG-15%**	6,432.3 ± 18.6
**MG-20%**	4,536.4 ± 30.8

Supplementary Table 12 | The tensile strength and toughness of the fabricated MGP fibers with different weight percentages of PVA.

Samples	Tensile strengths (MPa)/(CV)	Strain (%)/(CV)	Toughness (MJ m^{-3})/(CV)
**MG-5%**	335.6 ± 3.8 (0.01)	2.0 ± 0.1 (0.05)	4.0 ± 0.1 (0.03)
**MGP-1%**	393.6 ± 7.3 (0.02)	3.1 ± 0.1 (0.03)	6.8 ± 0.5 (0.07)
**MGP-2%**	458.3 ± 4.4 (0.01)	4.4 ± 0.2 (0.05)	9.4 ± 0.7 (0.07)
**MGP-5%**	565.2 ± 5.2 (0.01)	6.0 ± 0.1 (0.02)	19.2 ± 0.7 (0.04)
**MGP-10%**	315.6 ± 10.0 (0.03)	6.2 ± 0.3 (0.05)	9.7 ± 1.0 (0.10)
**MGP-15%**	196.0 ± 6.7 (0.03)	7.1 ± 0.3 (0.04)	8.4 ± 0.3 (0.01)
**PVA**	40.7 ± 2.2 (0.05)	235.3 ± 15.4 (0.07)	60.2 ± 2.2 (0.04)

Supplementary Table 13 | The electrical conductivity of the fabricated MGP fibers with different weight percentages of PVA.

Sample	Electrical conductivity (S cm^{-1})
**MG-5%**	9,860.6 ± 117.5
**MGP-1%**	9,350.3 ± 14.8
**MGP-2%**	9,003.9 ± 34.9
**MGP-5%**	8,110.4 ± 115.6
**MGP-10%**	5,258.7 ± 66.0
**MGP-15%**	3,966.8 ± 40.3

Supplementary Table 14 | The orientation order (*f*) and porosity of the fabricated MGP-T fibers via various draw-down ratios.

Samples	Orientation order (*f*)	Porosity (%)
**MGP-T_1**	0.844	8.6 ± 0.1
**MGP-T_2**	0.874	7.2 ± 0.1
**MGP-T_3**	0.891	5.7 ± 0.3

Supplementary Table 15 | The electrical conductivity of the fabricated MGP-T fibers via various draw-down ratios.

Sample	Electrical conductivity (S cm^{-1})
**MGP-T_1**	8,344.5 ± 23.4
**MGP-T_2**	8,555.2 ± 10.4
**MGP-T_3**	8,802.4 ± 30.8

Supplementary Table 16 | The tensile strength and toughness of the fabricated MGP-T fibers via various draw-down ratios.

**Samples**	**Tensile strengths (MPa)/(CV)**	**Strain (%)/(CV)**	**Toughness (MJ m^{-3})/(CV)**
**PC**	72.3 ± 3.7 (0.05)	44.1 ± 1.1 (0.02)	26.9 ± 0.9 (0.03)
**MGP-T_1**	372.5 ± 1.6 (0.01)	26.0 ± 0.5 (0.02)	77.9 ± 2.1 (0.03)
**MGP-T_2**	437.9 ± 6.5 (0.01)	21.4 ± 0.3 (0.01)	71.5 ± 4.3 (0.06)
**MGP-T_3**	585.5 ± 2.1 (0.01)	18.0 ± 0.5 (0.03)	66.7 ± 5.0 (0.07)

Supplementary Table 17 | The electrical conductivity retention rates under different loading-unloading cyclic testing conditions for MXene-based fibers.

**Loading stress (MPa)**	**Samples**	**Number of cycles**	**Electrical conductivity retention rate (%)**
**150**	MXene	5000	63.8
	MG	5000	75.5
	MGP	5000	90.3
	MGP-T	5000	98.9
**300**	MG	5000	64.2
	MGP	5000	81.3
	MGP-T	5000	93.5
**350**	MGP-T	5000	85.4

Supplementary Table 18 | Comparison of the tensile strength, toughness, and electrical conductivity of ultra-compact MXene fibers with that for other reported MXene-based fibers without protective layers.

Fibers	Fabrication method	Tensile strength (MPa)	Toughness ($MJ\ m^{-3}$)	Conductivity ($S\ cm^{-1}$)	Protective layer	Ref. No.
**MXene/Graphene**	Wet spinning	12.9	~0.2	290	No	19
**MXene/rGO**	Wet spinning	132.5	~0.3	72.3	No	23
**MXene/PEDOT:PSS**	Wet spinning	58.1	~0.3	1,489.8	No	42
**Pure MXene Fiber**	Wet spinning	63.9	~0.1	7,713	No	7
**LC MXene fiber**	Wet spinning	40.5	~0.4	7,750	No	15
**Pure $Ti_3C_2T_x$**	Wet spinning	118	~1.0	7,200	No	26
**Kevlar/$Ti_3C_2T_x$**	Wet spinning	18	~0.3	0.01128	No	21
**MXene/Cellulose**	Wet spinning	136.5	~3.4	4.8	No	20
**Pure MXene**	Wet spinning	343.68	~0.5	12,503.78	No	25
**rGO/MXene**	Wet spinning	110.7	3.8	743.1	No	43
**MXene/PU**	Wet spinning	7.8	-	1	No	44
**$Ti_3C_2T_x$/ANF**	Wet spinning	104	~4.1	1,025	No	37
**MXene Aerogel**	Wet spinning	~1.0	~0.01	117.09	No	45
**MXene/CNT**	Biscrolling	38.4	~1.0	2.7	No	46
**MXene/PEDOT:PSS-coated carbon tow**	Coating	~3000	-	~198	No	17
**MXene-coated cotton yarn**	Coating	460	-	199	No	17

**MGP**	**Wet spinning**	**565.2**	**19.2**	**8,110.4**	**No**	**This work**
**MGP-T_1**	**Wet spinning/Thermal drawing**	**372.5**	**77.9**	**8,344.5**	**Yes**	**This work**
**MGP-T_2**	**Wet spinning/Thermal drawing**	**437.9**	**71.5**	**8,555.2**	**Yes**	**This work**
**MGP-T_3**	**Wet spinning/Thermal drawing**	**585.5**	**66.7**	**8,802.4**	**Yes**	**This work**

Supplementary Table 19 | Comparison of the tensile strength, toughness, and electrical conductivity of ultra-compact MXene fibers with that for other reported carbon-based fibers.

Fibers	Tensile strength (MPa)	Toughness (MJ m^{-3})	Conductivity (S cm^{-1})	Protective layer	Ref. No.
**rGG-Ca^{2+}**	501.5	16.8	410	No	S3
**rGO-NaOH**	140.0	3.9	250	No	S4
**rGO-Ag-NW**	300.0	7.8	930	No	S5
**Porous rGO fiber**	50.0	0.3	25.1	No	S6
**Writing rGO**	350.0	5.6	270	No	S7
**LGO-SA**	784.9	6.8	35.8	No	S8
**Dry film scrolled**	40.0	0.3	416	No	S9
**rGO-chitosan**	115.0	1.4	2.8	No	S10
**rGO-CTAB**	175.0	3.2	35.0	No	S11
**CRG-PVA**	160.0	2.9	3.5	No	S12
**rGO-HPG**	165.0	1.2	4.9	No	S13
**rGO-NaDC**	238.0	2.4	308	No	S14
**rGO-Ca^{2+}-PCDO**	842.0	15.8	292.4	No	S15
**RGG-HPG-HI**	487.0	9.5	52.0	No	S16
**BGNF**	740.1	18.7	384.3	No	S17
**rGO-CS-Ca^{2+}**	743.6	26.3	179.0	No	S18
**Graphene fiber**	~1100	~8	1,100	No	6
**GF**	1450	~5.4	8,000	No	24
**Twisted MWNY fiber**	~300	~11.3	5,000	No	8

**Multilayered CNT fiber**	~700	~63	5,000	No	S19
**MGP**	**565.2**	**19.2**	**8,110.4**	**No**	**This work**
**MGP-T_1**	**372.5**	**77.9**	**8,344.5**	**Yes**	**This work**
**MGP-T_2**	**437.9**	**71.5**	**8,555.2**	**Yes**	**This work**
**MGP-T_3**	**585.5**	**66.7**	**8,802.4**	**Yes**	**This work**

Supplementary Table 20 | Comparison of the EMI performance, tensile strength, and toughness of ultra-compact MXene fibers with that of other reported MXene-based materials.

Material	Fabrication method	Test Type	Thickness (cm)	Conductivity (S cm^{-1})	*SE* (dB)	Strength (MPa)	Toughness (MJ m^{-3})	*SSEt* (×10^3 dB cm^{2}g^{-1})	Ref. No.
$Ti_3C_2T_x$	Freeze drying	Foam	6×10^{-3}	5800	70	4	-	53	48
$Ti_3C_2T_x$	Free-casted	Aerogel	0.2	~22	62	-	-	50	49
$Ti_3C_2T_x$	Bidirectional freeze-casted	Aerogel	0.1	-	70.6	-	-	64	49
$Ti_3C_2T_x$	Bidirectional freeze-casted	Aerogel	0.1	-	69.2	-	-	63	49
Ti_3CNT_x	Bidirectional freeze-casted	Aerogel	0.1	-	54.1	-	-	49	49
$Ti_3C_2T_x$/rGO	Bidirectional freeze-casted/Expoxy impregnated	Aerogel	0.2	7	56.4	-	-	-	49
$Ti_3C_2T_x$	Filtration	Film	0.45×10^{-3}	4800	92	-	-	-	13
$Ti_3C_2T_x$/SA	Filtration	Film	0.8×10^{-3}	2900	57	-	-	31	13
$Mo_2TiC_3T_x$	Filtration	Film	0.35×10^{-3}	250	26	-	-	-	13
SBM	Filtration	Film	0.3×10^{-3}	6115	56.4	583	15.9	62	S20
$Ti_3C_2T_x$/TOCNF	Filtration	Film	3.8×10^{-3}	283.7	32.7	141.9	1.7	4.761	S21
$Ti_3C_2T_x$/ANFs	Filtration	Film	1.7×10^{-3}	100	28.5	80.1	-	13	S22
MXene-CNT/PVA	Layer-by-layer assembly	Film	1.7×10^{-5}	130		~25	-	58	S23
MXene/MTM/PVA	Dip- Layer-by-layer assembly	Film	0.3×10^{-3}	-	20	225	-	25	S24

CF/MXene/MoS_2 fiber	Electrostatic adsorption /Hydrotherml	Bulk	3.5	-	~61.51	-	-	-	S25
Graphene/PS	Hot pressing	Film	0.25	-	45.1	110	-	0.692	S26
MCNT/PC	Solvent casting technique	Film	0.21	-	39	15	-	0.163	S27
Copper	-	-	0.31	-	-	-	-	32	13
Al	-	Foil	0.8×10^{-3}	-	-	-	-	31	13
Cu	-	Foil	1×10^{-3}	-	-	-	-	8	13
Stainless steel	-	-	0.4	-	-	-	-	0.028	S20
**MXene fiber**	**Wet spinning**	**Textile**	**~0.05**	**~11360.4**	**~75**	**167.0**	**0.4**	**48**	**This work**
**MGP fiber**	**Wet spinning**	**Textile**	**~0.05**	**~8110.4**	**~50**	**565.2**	**19.2**	**40**	**This work**
**MGP-T_3 fiber**	**Wet spinning/Thermal drawing**	**Textile**	**~0.05**	**~8,802.4**	**~61**	**585.5**	**66.7**	**43**	**This work**

Supplementary Table 21 | Comparison of the ET heating performance, tensile strength, and toughness of ultra-compact MXene fibers with that of other reported MXene-based fibers.

Material	Fabrication method	Diameter (mm)	Voltage (V)	Temperature (ºC)	Tensile strength (MPa)	Toughness (MJ m^{-3})	Protective layer	Ref. No.
MAFs fiber	Wet spinning/ supercritical drying	~0.420	4.5	178	1.1	-	No	45
MXene/Cellulose fiber	Wet spinning	~0.050	5<br>10<br>15<br>20	33<br>43<br>70<br>109	136.5	~3.4	No	20
MF fiber	Wet spinning	~0.012	33.6 (mA)	~60	343.68	~0.5	No	25
L-Ti_3C_2	Wet spinning	-	24	66	~40	-	No	S28
Pure MXene fiber	Wet spinning	~0.250	3<br>6<br>9<br>12	35<br>58<br>90<br>108	118	-	No	26
MXene/Aramid fiber	Wet spinning	0.158	3<br>9<br>15<br>21	25<br>35<br>54<br>123	160	-	Yes	S29
**MGP-T_3 fiber**	**Wet spinning /Thermal drawing**	**0.075**	**2**<br>**4**<br>**6**<br>**8**	**39**<br>**70**<br>**103**<br>**130**	**~585.5**	**~66.7**	**Yes**	**This work**<br>**This work**<br>**This work**<br>**This work**

Supplementary Table 22 | The flexibility of MXene, MG, MGP, and MGP-T fibers.

**Samples**	**Flexibility ($\times 10^8$ N^{-1} m^{-2})/(CV)**
**MXene**	0.62 ± 0.01 (0.02)
**MG**	1.25 ± 0.06 (0.05)
**MGP**	2.47 ± 0.12 (0.05)
**MGP-T**	12.91 ± 0.65 (0.05)

Supplementary Table 23 | The spray rate of the textiles prepared by manual weaving and machine weaving with MGP-T fibers.

**Type of weaving**	**Samples**	**Average rating**	**Comments**
**Manual weaving**	Textile	0	Complete wetting
	Textile+MGP-T fiber	0	Complete wetting
**Machine weaving**	Textile	70	Partial wetting
	Textile+MGP-T fiber	70	Partial wetting

Supplementary References

1. Peyttersson, T. et al. Measurement of the flexibility of wet cellulose fibres using atomic force microscopy. *Cellulose* **24**, 4139-4149 (2017).

2. He, F. et al. Ultraflexible Neural Electrodes for Long-Lasting Intracortical Recording. *iScience* **23**, 101387-101401 (2020).

3. Xu, Z. et al. Ultrastrong fibers assembled from giant graphene oxide sheets. *Adv. Mater.* **25**, 188-193 (2013).

4. Xu, Z. & Gao, C. Graphene chiral liquid crystals and macroscopic assembled fibres. *Nat. Commun.* **2**, 571 (2011).

5. Xu, Z. et al. Highly electrically conductive Ag-doped graphene fibers as stretchable conductors. *Adv. Mater.* **25**, 3249-3253 (2013).

6. Aboutalebi, S. H. et al. High-performance multifunctional graphene yarns: toward wearable all-carbon energy storage textiles. *ACS Nano* **8**, 2456-2466 (2014).

7. Cao, J. et al. Programmable writing of graphene oxide/reduced graphene oxide fibers for sensible networks with in situ welded junctions. *ACS Nano* **8**, 4325-4333 (2014).

8. Hu, X. et al. A novel wet-spinning method of manufacturing continuous bio-inspired composites based on graphene oxide and sodium alginate. *Nano Research* **9**, 735-744 (2016).

9. Cruz-Silva, R. et al. Super-stretchable graphene oxide macroscopic fibers with outstanding knotability fabricated by dry film scrolling. *ACS Nano* **8**, 5959-5967 (2014).

10. Jalili, R. et al. Scalable one-step wet-spinning of graphene fibers and yarns from liquid crystalline dispersions of graphene oxide: towards multifunctional textiles. *Adv. Fun. Mater.* **23**, 5345-5354 (2013).

11. Cong, H. et al. Wet-spinning assembly of continuous, neat, and macroscopic graphene fibers. *Sci. Rep.* **2**, 613 (2012).

12. Kou, L. & Gao, C. Bioinspired design and macroscopic assembly of poly(vinyl alcohol)-coated graphene into kilometers-long fibers. *Nanoscale* **5**, 4370-4378 (2013).

13. Hu, X. & Gao, C. Multifunctional, supramolecular, continuous artificial nacre fibres. *Sci. Rep.* **2**, 767 (2012).

14. Huang, G. et al. Highly strong and elastic graphene fibres prepared from universal graphene oxide precursors. *Sci. Rep.* **4**, 4248 (2014).

15. Zhang, Y. et al. Ultrastrong bioinspired graphene-based fibers via synergistic toughening. *Adv. Mater.* **28**, 2834-2839 (2016).

16. Hu, X. et al. Liquid crystal self-templating approach to ultrastrong and tough biomimic

composites. *Sci. Rep.* **3**, 2374 (2013).
17. Zhang, Y. et al. Bioinspired supertough graphene fiber through sequential interfacial interactions. *ACS Nano* **12**, 8901-8908 (2018).
18. Wang, X. et al. Ultratough bioinspired graphene fiber via sequential toughening of hydrogen and ionic bonding. *ACS Nano* **12**, 12638-12645 (2018).
19. Zhong, X. et al. Continuous multilayered carbon nanotube yarns. *Adv. Mater.* **22**, 692-696 (2010).
20. Wan, S. et al. High-strength scalable MXene films through bridging-induced densification. *Science* **374**, 96-99 (2021).
21. Zhan, Z. et al. Ultrastrong and conductive MXene/cellulose nanofiber films enhanced by hierarchical nanoarchitecture and interfacial interaction for flexible electromagnetic interference shielding. *J. Mater. Chem. C* **7**, 9820-9829 (2019).
22. Xie, F. et al. Ultrathin MXene/aramid nanofiber composite paper with excellent mechanical properties for efficient electromagnetic interference shielding. *Nanoscale* **11**, 23382-23391 (2019).
23. Weng, G. et al. Layer-by-layer assembly of cross-functional semi-transparent MXene-carbon nanotubes composite films for next-generation electromagnetic interference shielding. *Adv. Funct. Mater.* **28**, 1803360-1803368 (2018).
24. Lipton, L. et al. Mechanically strong and electrically conductive multilayer MXene nanocomposites. *Nanoscale* **11**, 20295-20300 (2019).
25. Wang, J. et al. Hierarchical carbon fiber@MXene@MoS_2 core-sheath synergistic microstructure for tunable and efficient microwave absorption. *Adv. Funct. Mater.* **30**, 2002595-2002604 (2020).
26. Yan, D. et al. Structured reduced graphene oxide/polymer composites for ultra-efficient electromagnetic interference shielding. *Adv. Funct. Mater.* **25**, 559-566 (2015).
27. Pande, S. et al. Mechanical and electrical properties of multiwall carbon nanotube/polycarbonate composites for electrostatic discharge and electromagnetic interference shielding applications. *RSC Adv.* **4**, 13839-13849 (2014)
28. Naguib, M. Multifunctional pure MXene fiber from liquid crystals of only water and MXene. *ACS Cent. Sci.* **6**, 344-346 (2020).
29. Wang, L. et al. Lightweight, robust, conductive composite fibers based on MXene@Aramid nanofibers as sensors for smart fabrics. *ACS Appl. Mater. Interfaces* **13**, 41933-41945 (2021).
