# Supplementary material for: Ultra-compact MXene fibers by continuous and controllable synergy of interfacial interactions and thermal drawing-induced stresses
Source: Nat Commun. 2022 Aug 5;13:4564. doi: 10.1038/s41467-022-32361-6 (PMC9356020; doi:10.1038/s41467-022-32361-6)
Supplement: Supplementary file 2 — Description of Additional Supplementary Files [file 41467_2022_32361_MOESM2_ESM.pdf]

**Description of Additional Supplementary Files**

File Name: Supplementary Movie 1
Description: The preparation process of MGP fibers via wet spinning. The video shows that several meters long MGP fibers can be fabricated with a continuous and controllable method.

File Name: Supplementary Movie 2
Description: The preparation process of ultra-compact MGP-T fibers by thermal drawing. The video shows that the MGP fibers can be continuously fabricated with several meters long, resulting in the formation of an outer protective layer for the core of MGP fibers.

File Name: Supplementary Movie 3
Description: Finite element analysis for thermal drawing process. The video shows that the PC hollow tube model is stretched with the reduction of the diameter of the PC hollow tube. Moreover, when increasing the draw-down ratio in simulation, Z compressing stress is increased, according to the results of finite element analysis.

File Name: Supplementary Movie 4
Description: A white sweater woven with several ultra-compact MGP-T fibers generates heat at the applied DC voltage of 4 V. The video shows that a sweater with several MGP-T fibers could quickly generate the heat with the temperature of ~70 ºC for wearable human thermal management.

File Name: Supplementary Movie 5
Description: Electrothermal heat bending stability of the textile weaved by the ultra-compact MGP-T fibers at the applied DC voltage of 4 V. The video shows that the textile with several MGP-T fibers shows excellent stability when bending for wearable human thermal management.
